# Assessing Obesogenic School Environments in Sibiu County, Romania: Adapting the ISCOLE School Environment Questionnaire

**DOI:** 10.3390/children10111746

**Published:** 2023-10-27

**Authors:** Mihai Octavian Negrea, Gabriel Octavian Negrea, Gabriela Săndulescu, Bogdan Neamtu, Raluca Maria Costea, Minodora Teodoru, Călin Remus Cipăian, Adelaida Solomon, Mirela Livia Popa, Carmen Daniela Domnariu

**Affiliations:** 1 Medical Clinical Department, Faculty of Medicine, “Lucian Blaga” University, 550024 Sibiu, Romania; mihaioctavian.negrea@ulbsibiu.ro (M.O.N.); bogdan.neamtu@ulbsibiu.ro (B.N.); minodora.teodoru@ulbsibiu.ro (M.T.); calin.cipaian@ulbsibiu.ro (C.R.C.); adelaida.nuta@ulbsibiu.ro (A.S.); liviamirela.popa@ulbsibiu.ro (M.L.P.); 2 County Clinical Emergency Hospital of Sibiu, 2–4 Corneliu Coposu Str., 550245 Sibiu, Romania; 3 “Gheorghe Lazăr” National College, 1–3 Gheorghe Lazăr Str., 550165 Sibiu, Romania; gabriela.sandulescu@cnglsibiu.ro; 4 Department of Clinical Research, Pediatric Clinical Hospital Sibiu, 550166 Sibiu, Romania; ralucacosteadr@gmail.com; 5 Pediatric Neurology Department, Pediatric Clinical Hospital Sibiu, 550166 Sibiu, Romania; 6 Department of Dental Medicine and Nursing, Faculty of Medicine, “Lucian Blaga” University, 550024 Sibiu, Romania; carmen.domnariu@ulbsibiu.ro

**Keywords:** childhood obesity, school environment, physical activity, healthy eating, public health policies, rural schools

## Abstract

The impact of the school environment on childhood weight status has garnered significant attention in recent years. This study aimed to adapt and validate the International Study of Childhood Obesity, Lifestyle and the Environment (ISCOLE) School and Environment questionnaire in order to assess the potential obesogenic impact of school environments in Sibiu County, Romania. The ISCOLE questionnaire was chosen for its rigorous methodology. It was derived from a comprehensive study conducted across 12 countries which aimed to capture multifaceted influences on childhood weight while emphasizing educational settings in the collection of data. To guide the translation and adaptation of the questionnaire, a multidisciplinary committee was assembled which comprised experts in teaching and school administration to ensure target responder relevance, experts in clinical research to ensure methodological robustness, experts in language adaptation to preserve the original intent of the survey, and experts in public health to steer the interpretation of the results, with potential policy implications. The data were analyzed by distinguishing between urban and rural settings, and a two-step cluster analysis was implemented to identify potential intervention targets. To assess the validity of the adapted tool, the questionnaire’s construct validity and internal consistency were explored. A response rate of 71.2% of the approached schools in Sibiu County was achieved. Of the 84 responding school representatives, 37 (44%) were from a rural setting. The rural schools had significantly more limited access to gymnasiums, secured lockers, showers, and bicycle racks, and exhibited more serious problems regarding the inadequate disposal of garbage in the school vicinity. A two-step cluster analysis revealed distinct school categories, providing opportunities for public policy interventions. One of these primarily concerned rural schools with limited infrastructure but with proactive practices and policies which were termed “unable but willing”; on the opposing spectrum, the category “able but unwilling” mainly comprised urban schools which had available facilities but lacked local proactive initiatives. The findings emphasize the urgent need for targeted measures to bridge these discrepancies by investing in infrastructure in rural schools and promoting active school practices and policies in urban settings. The assessment of obesogenic school environments in Sibiu County provides a pilot model for broader applications due to the diverse school landscape and supportive local authorities. The results, which were achieved using low-cost methods, can guide future educational policies, health promotion initiatives, and preventive interventions.

## 1. Introduction

The escalating incidence of childhood obesity is a pressing global issue, with consequences that extend from immediate health hazards to long-term complications, notably increased risks of adult obesity and cardiovascular diseases [1,2,3,4].

Childhood obesity can indirectly diminish physical and cognitive performance, as highlighted by the adverse effects of sleep deprivation on student athletes’ abilities [5]. Additionally, significant variations in anthropometric traits among high school students underline the urgency of addressing lifestyle factors influencing youth, especially amid modern sedentary challenges [6]. The causative mechanisms of obesity are complex and multifaceted, as discussed in our previous work [7]. They are influenced not just by caloric imbalance but also a myriad of individual, societal, and environmental factors ranging from overarching elements, such as a country’s economic health, to the closer influences of the adults with whom children live [8] or finer intricacies such as an individual’s self-perception. In these circumstances, a social–ecological approach is required to understand and address influences on childhood weight status [9]. School environments are pivotal in this discourse.

In the EU and the United States, children spend a substantial part of their day in school [10,11]. This makes schools a significant arena for potential health interventions, as the habits formed during this time are foundational and likely to be carried into adulthood [12,13,14]. Despite existing health guidelines from bodies such as the CDC, which advocate specific benchmarks for physical activity and sleep duration for children and adolescents [15,16], these standards are frequently unachieved. This discrepancy is partly attributable to the predominantly sedentary nature of school activities and the challenge of juggling academic and extracurricular demands with adequate rest [17,18,19,20,21].

Numerous elements can sway student health outcomes in a school setting, particularly concerning nutrition and physical activity. It is imperative for public health policies to pinpoint these variables and strive to attenuate detrimental influences while amplifying positive ones. For a detailed insight, Appendix A enumerates instances of school-level exposures pertaining to nutrition and physical activity; these instances are accompanied by an analysis of their obesity-related impacts which is supported by the pertinent literature and suggests potential policy interventions for risk mitigation and the promotion of health. Persistent efforts have been directed toward meticulously evaluating the principal correlates of dietary trends and physical activity among children and adolescents, exemplified by The International Study of Childhood Obesity, Lifestyle and the Environment (ISCOLE). ISCOLE’s multi-national inquiry into the lifestyle and environmental correlates of childhood obesity yielded invaluable methodological insights, especially regarding data procurement and handling [22].

In particular, concerning school-related exposures, data were collected via a questionnaire addressed to school administrations; this questionnaire was then doubled via an audit performed by an instructed member of the study’s staff. In the ISCOLE study, data sampling was conducted in school clusters, thus positioning the educational setting as a pivotal factor in organizing and interpreting the collected data [22,23].

The main objective of the present study was to adapt the school environment questionnaire used in the ISCOLE study to the specific setting of Romanian schools. To achieve this goal, a research partnership was established between the Pediatric Clinical Hospital of Sibiu, a local high school in Sibiu, and the Sibiu County Board of Education in order to conduct this community-oriented research project. The study also expanded the research to include rural schools, providing a more comprehensive view of the influences on children’s health in rural and urban settings.

## 2. Materials and Methods

### 2.1. Adapting the Questionnaire

The translation and adaptation of the questionnaire aimed to maintain the validity of the initial instrument. A series of guidelines were used to govern the process of culturally adapting validated questionnaires in healthcare [24,25]. As such, the steps described below were undertaken.

#### 2.1.1. Expert Committee Assembly

In order to culturally adapt the ISCOLE School Environment questionnaire, a research partnership was established between the Research Department of the Pediatric Clinical Hospital of Sibiu, the “Gheorghe Lazăr” National College of Sibiu, and the Sibiu County Board of Education, forming a team comprising members of the school administration, pediatricians, experienced researchers, cardiologists, and other clinicians. The collaboration within the study also extended to members of the Medicine Faculty of the “Lucian Blaga” University of Sibiu and included an expert in the field of public health. In addition, the study employed the services of a local authorized translation bureau, as well as an independent native Romanian with expert English knowledge (the individual holds a Ph.D. in English and is an assistant professor of English at Shaw University, North Carolina).

#### 2.1.2. Adaptation and Forward Translation

Adapting the questionnaire to local circumstances was necessary to improve its effectiveness. This was carried out by the first author (M.O.N., Ph.D. student and resident doctor in cardiology) in collaboration with the principal (G.O.N.) and assistant principal (G.S.) of the “Gheorghe Lazăr” National College of Sibiu. The forward/backward translation process is a commonly implemented and validated method for ensuring adequate concept equivalence [26].

The forward translation was carried out by M.O.N. and G.O.N. The translation was further refined to enhance the clarity of the questions and the feasibility of the study by B.N., R.M.C. (Department of Clinical Research, Pediatric Clinical Hospital Sibiu), C.D.D. (university professor of public health, “Lucian Blaga” University of Sibiu), and G.S.

#### 2.1.3. Backward Translation

A backward translation was achieved by employing two legally authorized translators from a local translation firm. The original questionnaires, as well as the scope of the study, were not revealed to the translators.

#### 2.1.4. Translation Review

The initial questionnaire, the forward translation, and the backward translation were reviewed by a third party, a native Romanian proficient in English (who holds a Ph.D. in English and is an assistant professor of English at Shaw University, North Carolina) to ensure that the meaning of each question was adequately retained.

### 2.2. Data Collection

In a similar fashion to the original ISCOLE school environment questionnaire, the collected data were comprised of inquiries covering school characteristics, policies and practices, physical activity within the school environment, school facilities, healthy eating practices, and school neighborhood characteristics [22].

A detailed overview of each question from the ISCOLE school environment questionnaire, as well as their forward- and backward-translated versions, can be found in the Supplementary Materials, Table S1. A changelog describing all the modifications performed on the questionnaire is available in Appendix B. The original unrevised backward translation, as well as the official statements from both of the official translators, can be found in the Supplementary Materials, Scan S1.

The questionnaires were administered through Google Forms, a web-based data collection tool, to facilitate centralized data gathering and storage between June and July 2023. A first call to answer the questionnaires was issued to the headmasters of the schools registered in the county of Sibiu by the County Board of Education on the 8th of June.

This study adopted a comprehensive inquiry approach wherein the target population encompassed all accessible school administrations within the defined geographic region. This approach was guided by the principle of exhaustive sampling within the context and constraints of our study, with the aim of capturing a holistic and extensive array of perspectives and policies prevalent in these educational institutions.

A database of educational institutions within Sibiu County provided by the County Board of Education was used to gauge the response rate. A second call, which also referred to the names of the institutions from which no response had been received, was issued on the 13th of July to improve the response rate. The same database used to measure the response rate also served to document the schools’ urban or rural environment location, which was self-reported by each school administration. Duplicate or incomplete answers were excluded from the data analysis.

### 2.3. Pilot Sample

**Figure 1:**
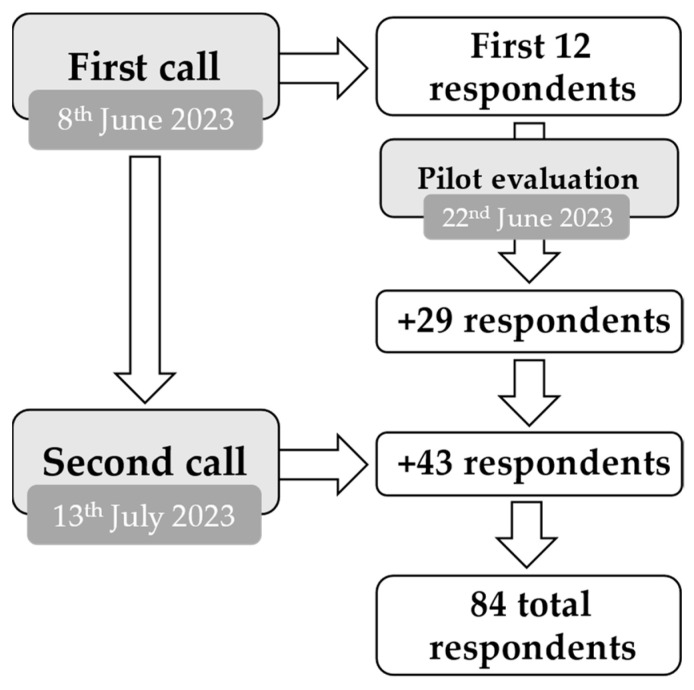
Sample recruitment process.

After issuing the first call on the 8th of June, the first 12 responses were evaluated by the expert committee to ensure the clarity and comprehension of the questions and to identify potential problems that could arise during the collection of data. The number of respondents for the pilot test was established according to the guiding literature in this regard [27,28]. Particularly, in his comprehensive work on the construction of questionnaires which established him as an expert in this field, Sheatsley P. stated, based on his extensive research, “it usually takes no more than 12–25 cases to reveal the major difficulties and weaknesses in a pretest questionnaire” [29]. Moreover, in the current study, 12 cases represented more than 10% of the maximum potential respondents (there are 118 schools registered in the County of Sibiu), thus providing an adequate pilot sample size. As no issues were identified, data collection commenced with no further modifications to the methodology or the questionnaires. The sample recruitment process is depicted in Figure 1.

### 2.4. Statistical Analysis

Analyses of categorical variables were depicted using frequencies and percentages. Where suitable, 95% confidence intervals (95%CIs) were computed for proportions based on the marginal error $E=z\sqrt{p\left(1-p\right)/n}$, where z=1.96, the z value for 95% confidence intervals, n is the sample size, and p is the proportion in question. Accordingly, 95% CI values were defined as 95%CI upper limit=p+E and 95%CI lower limit=p−E. When the 95%CI limits either exceeded 100% or fell below 0%, they were truncated at these thresholds. Continuous variables were described using means, standard deviations, minimum and maximum values, interquartile ranges, and 95% confidence intervals for means. The normality of continuous variables was assessed using the Kolmogorov–Smirnov or Shapiro–Wilk tests. To compare groups, the chi-squared or Fischer’s exact tests were utilized for categorical variables, and the independent *t*-test was applied to continuous variables following a normal distribution when comparing means across two independent groups. For continuous variables without a normal distribution, the Mann–Whitney U test was employed. When means were compared across three or more categories, a one-way ANOVA test was used for normally distributed variables, and the Kruskal–Wallis test was used for skewed distributions. The significance threshold was set at an α-level of 0.05.

This study utilized a two-step clustering method to investigate groupings among characteristics relating to school size, attitudes toward physical activity, and the availability of physical-activity-related facilities. The two-step clustering technique operates in two main stages. Initially, the method sifts through the dataset, forming multiple small sub-clusters by gauging the similarity between data points. This procedure is similar to the k-means clustering approach, which aims to keep data points as close to their cluster’s centroid as possible. In the second step, hierarchical clustering is applied, which sequentially merges the most similar data points to form increasingly larger clusters.

The optimal number of clusters and the best-fitting model can be determined using Akaike’s information criterion (AIC), which balances a model’s fit and complexity. Additionally, the Silhouette Score is used to evaluate how well an object aligns with its designated cluster in comparison to others. Two-step clustering algorithms present distinct advantages: firstly, they adeptly handle both categorical and continuous variables by assuming their independence, facilitating the placement of a joint multinomial–normal distribution. Secondly, they can autonomously determine the optimal number of clusters by evaluating the values of a model-choice criterion across different clustering solutions.

In this research, Akaike’s information criterion was employed to determine the best number of clusters. The focus was oriented toward variables related to school size, practices and policies concerning physical activity, and school facilities for physical activity. A good average silhouette value of cohesion and separation (greater than 0.5) was targeted. Furthermore, predictor variables were retained in the model only if they had a predictor importance of at least 0.5.

### 2.5. Questionnaire Validation

The construct validity and internal consistency of the questionnaire were explored using items with identical non-binary response scales for which the respondents’ perceptions may have influenced their answers. The questionnaire’s construct validity was evaluated using a principal component analysis on appropriate items. After initially determining the loading of each item, a varimax rotation was applied. This rotation optimized the loading of each item on the extracted components, helping to clarify the structure of the data. The questionnaire’s internal consistency was assessed by computing Cronbach’s alpha, using items which measured unique constructs identified during the PCA.

## 3. Results

Out of a total of 118 schools registered in the County of Sibiu, 84 answered the adapted questionnaire (a 71.2% response rate). Responses were provided by school administrators in 89.3% of cases (81% of the respondents were the schools’ principals, and 8.3% were the schools’ assistant principals), while 10.7% of the responses were provided by teachers working in the schools.

### 3.1. School Characteristics

**Figure 2:**
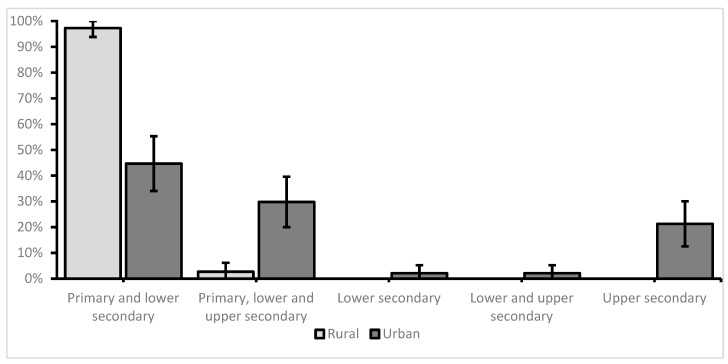
Education levels provided by the schools included in the study (percentages and 95%CIs)—Fischer’s exact test *p* < 0.01.

Of the participating schools, 37 (44%) were from a rural environment, while 47 (56%) were from an urban setting. The details regarding the schools’ characteristics are presented in Figure 2 and Table 1.

### 3.2. School Policies and Practices Regarding Healthy Eating and Physical Activity

Data regarding the allocation of hours of the curriculum per each school’s decision (CDS) dedicated to physical activity or healthy eating are presented in Table 2.

The curriculum according to each school’s decision is a component of the national curriculum, which is customized by individual schools to cater to their students’ specific educational needs. While the CDS emphasizes optional subjects and fields of study that can be adopted either at the national level or by particular schools, there is a mandated number of hours allotted for the CDS based on each educational level and pathway. The CDS can encompass between one and six periods weekly, contingent on the educational tier. The school determines the subjects or educational activities for these periods. In contrast, the Ministry of Education prescribes the remainder of the subjects and their respective weekly periods.

Results regarding the presence of specific policies and practices concerning physical activity and healthy eating are presented in Table 3.

Collectively, 72.6% of the participants confirmed the implementation of both physical activity and healthy eating policies or practices within their institution, namely 27 (73%) rural schools and 34 (72.3%) urban schools, with no statistical difference discernible between them (*p* = 1).

The policies regarding physical activity included access to indoor (10 respondents) or outdoor facilities (19 respondents) outside of school hours or the provision of sports equipment during recess (10 respondents), while the practices included various local and international projects and collaborations (4 respondents), extracurricular training (8 respondents) and classes (7 respondents), and field trips (22 respondents) or the organization of camps (5 respondents) and special events (7 respondents) or competitions (22 respondents). Policies concerning healthy eating were confined to specific food restrictions, while practices in this regard included the organization of special events or competitions and classes or workshops for healthy cooking, as well as various local and international collaborations.

Regarding the presence of committees tasked with addressing proposals related to physical activity or healthy eating, 36.2% of urban schools reported having such a committee compared to 16.2% of rural schools. This disparity was statistically significant (*p*-value = 0.042).

### 3.3. Physical Activity in School

**Figure 3:**
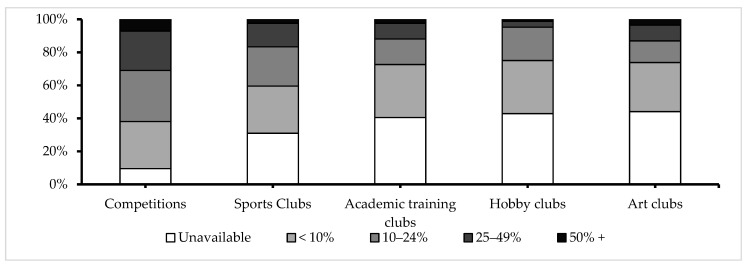
Extracurricular activities.

**Figure 4:**
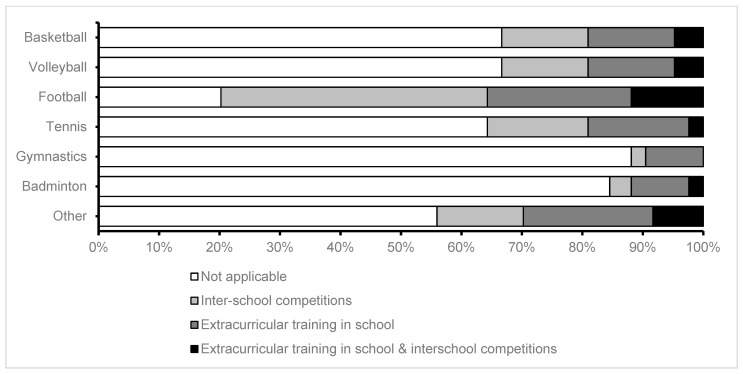
Extracurricular sports.

The most frequent extracurricular activities were interschool competitions (detailed in Figure 3), and the most frequently practiced sport was football (described in Figure 4). There were no statistically significant differences in the distributions of the frequencies regarding extracurricular activities or sports played between urban and rural environments.

The “other” category comprised handball (11 respondents), chess (8 respondents), table tennis (7 respondents), dancing (6 respondents), athletics (5 respondents), martial arts (3 respondents), oina (a traditional Romanian sport similar to baseball, 3 respondents), skiing (2 respondents), cycling (2 respondents), ecotourism (2 respondents), inline skating (1 respondent), karting (1 respondent), and fencing (1 respondent).

Data regarding the provision of transportation to extracurricular activities are presented in Table 4.

Concerning extended breaks, 44.05% of the participants indicated they had no breaks lasting between 15 and 29 min during school hours. Meanwhile, 47.62% reported having one break of this duration, and 8.33% mentioned having three or more breaks in this range. Only 3.57% stated they had a break exceeding 30 min during school hours. No statistically significant differences were found between rural and urban environments.

**Figure 5:**
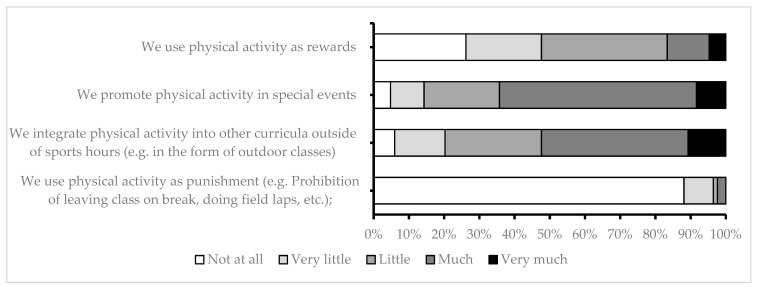
Attitudes toward physical activity.

School attitudes toward physical activity are presented in Figure 5. There were no statistically significant differences between rural and urban environments.

**Figure 6:**
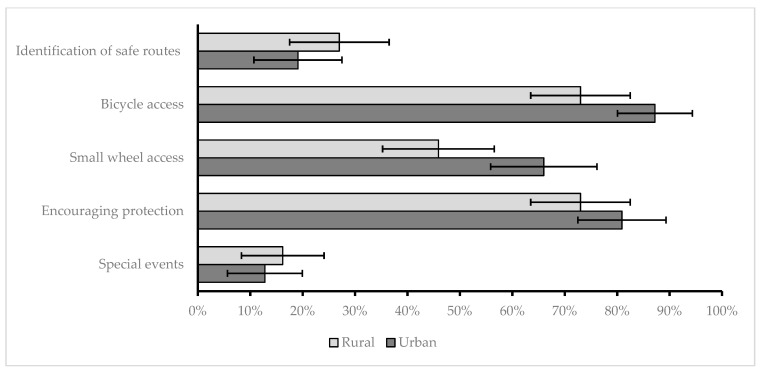
Promoting active transportation (error bars representing 95%CIs for the proportion of positive answers).

School attitudes toward promoting active transportation are presented in Figure 6.

### 3.4. School Facilities

The school facilities available to students for physical activity are presented in Table 5.

Overall, 85.71% of the responding schools had access to some form of large indoor space for practicing physical activities (gym/other large halls or spaces), and 98.8% of the respondents had access to some form of outdoor space intended for this purpose (running track/outdoor sports ground/paved area/lawn-covered area/fixed equipment playground).

Of the 67 respondents with access to a gym during school hours, 36 (53.7%) also noted providing extracurricular access to this facility, with no statistically significant differences between environments.

Extracurricular access to outdoor facilities was provided by 72.6% of the respondents, access to sports equipment was provided by 48.8% of respondents, and extracurricular access to school facilities dedicated to physical activity for organized groups was provided by 67.9% of the respondents. There were no significant differences between rural and urban settings regarding these aspects.

Data regarding eating facilities are presented in Table 6.

A total of 66.7% of the respondents exhibited at least one source of potential competitive food exposure by means of easy access to facilities providing unregulated foods, namely stores near the school, fast-food restaurants, and vending machines, with urban schools being more exposed to fast-food restaurants and drink-vending machines. Of the eight institutions with access to a cafeteria, seven had offered healthy food options in the previous 12 months. Of the seven schools with an in-school store, five had offered healthy food options in the previous 12 months.

### 3.5. Healthy Eating

The eating activities linked to healthy nutrition offered by the respondent schools are presented in Table 7.

### 3.6. School Surroundings

Issues relating to school surroundings across rural and urban environments are presented in Table 8.

### 3.7. Two-Step Cluster Analysis

A two-step cluster analysis employing Akaike’s information criterion was conducted. The variables included were the number of students and teaching positions as markers of school size, the presence of practices or policies implemented within the school, and access to a gym during school hours. This resulted in a model that defined four clusters with an average silhouette of cohesion and separation of 0.7, indicating good model quality. The characteristics of the model and the clusters obtained are presented in Table 9.

The distribution of environments across clusters is presented in Table 10.

Four clusters emerged from the analysis. Cluster 1 comprised smaller schools, most of which had implemented some form of policy or practice regarding physical activity but did not have access to a gym. In contrast, Cluster 2 consisted of small- and medium-sized schools that did have access to a gym but did not implement any practices or policies regarding physical activity. Most of the schools in Cluster 1 were from a rural environment, while the majority of schools in Cluster 2 were from an urban setting. Clusters 3 and 4 contained schools that both implemented practices or policies regarding physical activity and had access to a gym. They differed in size and environment, however, such that Cluster 3 primarily contained large schools from mostly urban environments, and Cluster 4 contained small- and medium-sized schools with a relatively uniform distribution across urban and rural settings.

### 3.8. Questionnaire Validation

A principal component analysis (PCA) was employed to evaluate the construct validity of questionnaire items that had identical non-binary measurement scales and which might be influenced by the respondents’ perspectives. Subsequently, Cronbach’s alpha was computed for these items to assess their internal consistency. Based on these criteria, three questions from the original questionnaire were deemed suitable for analysis: Questions 9, 16 and 27. In the adapted version of the questionnaire, these became Questions 13, 19 and 27.

For Question 13, which probed the participants’ levels of involvement in various extracurricular activities, the PCA extracted a single principal component. The first item, which addressed participation in interschool sports competitions, explained 50.55% of the variance in the data, with an eigenvalue of 2.527. When the second item, which focused on participation in school sports clubs/courses, was added, the cumulative variance explained rose to 70.09%. This second item had an eigenvalue of 0.977. In contrast, items related to participation in academic, hobby, or art clubs contributed less to the total variance and had eigenvalues below 1. An internal consistency analysis of Question 13 yielded a Cronbach’s alpha value of 0.744.

For Question 19 (originally Question 16), which sought to determine attitudes toward physical activity, the PCA extracted two components. The first item pertaining to the use of physical activity as a reward explained 45.08% of the variance, with an eigenvalue of 1.803. The second item, which addressed the promotion of physical activity during special events, explained an additional 25.01% of the variance, with an eigenvalue of 1.000, bringing the cumulative variance explained by the two components to 70.09%. The third and fourth items, which measured the integration of physical activity within the classroom and the use of physical activity as punishment, were observed but not extracted for a further analysis as they contributed 18.49% and 11.42% to the variance and had eigenvalues of less than 1. Before varimax rotation, the component matrix yielded that the first three items loaded significantly on component 1 with values of 0.673, 0.823, and 0.790, while item 4 mainly loaded on component 2 with a value of 0.953.

After varimax rotation, the loadings became 0.636 for item 1, 0.853 for item 2, and 0.802 for item 3 on component 1, while item 4 continued to load on component 4 with a value of 0.977. The Component Transformation Matrix showed that the two extracted components had values proximate to 1, which signifies that the rotation maintained most of the variance from the original components.

As these results indicated that items 1–3 and item 4 essentially measured different constructs, Cronbach’s alpha was computed only for items 1–3 and yielded a value of 0.643.

For Question 27, which inquired about the possible challenges a school might confront in its surroundings, the PCA extracted two components.

The first two items, which pertained to tensions based on ethnic, religious, or regional differences and garbage dumped in the school’s surroundings, explained 70.621% of the total variance. The first item had an eigenvalue of 4.628, explaining 57.853% of the variance, while the second item’s eigenvalue was 1.021, adding another 12.768%.

Before varimax rotation, all items except “heavy road traffic” loaded primarily onto component 1. Specifically, the loadings were: 0.768 for ethnic tensions, 0.728 for the presence of garbage, 0.783 for the sale of alcohol, 0.831 for drug usage, 0.888 for gangs, 0.720 for abandoned buildings, and 0.865 for the crime rate. The item concerning heavy traffic predominantly loaded onto component 2, with a value of 0.765.

After rotation, the loading values for component 1 were 0.717 for ethnic tensions, 0.834 for garbage, 0.825 for the sale of alcohol, 0.756 for drug usage, 0.840 for gangs, and 0.740 for the crime rate. For component 2, the loadings were 0.858 for heavy road traffic and 0.686 for abandoned buildings.

The computation of Cronbach’s alpha, which included all the items in Question 27, yielded a value of 0.882. However, when the item assessing heavy traffic was excluded, this value increased to 0.903.

## 4. Discussion

This study adapted and validated the ISCOLE School and Environment questionnaire for use in the setting of Romanian schools.

A meticulous methodology was employed to uphold the validity of the initial questionnaire. In this respect, a committee of experts in teaching, school administration, clinical research, public health, and language adaptation was formed. This committee oversaw the forward translation of the questionnaire to ensure its accuracy and fidelity with respect to the original content. Subsequently, an independent backward translation was conducted to validate that the essence and meaning of the questionnaire items were preserved. After the collection of data, the validity of the questionnaire was further tested by measuring its construct validity and internal consistency on appropriate items, which yielded adequate results.

The present study had a satisfactory response rate of 71.2% among the schools in Sibiu County. A total of 84 school representatives, 44% of which were from a rural setting, answered the adapted questionnaire.

Significant differences were observed between the schools situated in rural versus urban settings. Concerning school characteristics, the institutions in rural areas tended to be smaller with respect to the number of students and faculty, and were mostly oriented toward primary and lower secondary education.

Several studies have utilized the ISCOLE questionnaire to investigate the obesogenic influence of school environments, primarily focusing on the original ISCOLE study’s target demographic: children aged 9–11 years [23]. A noteworthy example is the work by Gomes et al. [30], which delved into school-level impacts on sedentary behavior. Their findings suggested that individual and family-related factors are more important in this regard.

In contrast, Crooks et al. [31] discerned a significant association between girls’ adherence to moderate-to-vigorous physical activity (MVPA) guidelines and factors such as the presence of physical activity policies and the availability of indoor or outdoor play areas. In a different vein, de Moraes Ferrari et al. [32] identified a significant correlation between boys’ body mass index and the implementation of school policies or practices advocating healthy eating. Sales et al. [33] drew connections between the duration of MVPA and various elements: the incorporation of physical-activity-focused policies or practices, the presence of a committee dedicated to the proposal and enactment of policies on physical activity or healthy eating, extended break periods, participation in interschool competitions, and access to outdoor sports facilities.

The present study, which leveraged a culturally adapted version of the ISCOLE questionnaire to evaluate school environments, produced findings that resonate with the above-mentioned research. This was especially evident in aspects related to the presence of policies or practices geared toward physical activity or healthy eating and the accessibility of facilities and activities underscoring these principles.

In this study, a high proportion of schools had some form of policy or practice regarding physical activity and healthy eating. More so, several initiatives stood out as noteworthy examples of innovative approaches to physical activity and healthy eating practices, and are presented in more detail in Appendix C.

When analyzing the allocation of hours of the CDS toward these two aspects, a more pronounced emphasis on healthy eating over physical activity was observed. There was a low prevalence of specialized committees regarding the implementation of practices and policies on physical activity and healthy eating, particularly in the rural environment. Further disparities between rural and urban schools were highlighted concerning access to a gymnasium, secured lockers, showers before and after physical activity, and bicycle racks. In all the aforementioned cases, rural schools were significantly less likely to benefit from these facilities. An additional point of concern was the issue of the disposal of garbage around school premises, which was significantly more prevalent in rural areas.

Furthermore, the accessibility of unregulated competitive foods in both rural and urban school settings is alarming, as two-thirds of the surveyed schools were in proximity to at least one such source, with urban schools being more susceptible to exposure to fast-food restaurants and drink-vending machines. These results showcase several potential areas in which public policy interventions could prove beneficial.

A two-step cluster analysis was performed to further refine the identification of potential targets for such interventions. The algorithm was based on school size, the enactment of practices or policies regarding physical activity, and access to school gymnasiums.

This approach resulted in four distinct school clusters within the studied sample. Clusters 3 and 4 consisted of either large or small-to-medium-sized schools which were characterized by having both access to an indoor gymnasium and established policies or practices on physical activity. These schools were labeled as “able and willing”.

Of particular interest, however, were two clusters that highlighted clear opportunities for public policy interventions. Cluster 1 encompassed smaller schools that lacked a gymnasium despite having proactive physical activity practices or policies. In contrast, Cluster 2 comprised larger schools with gymnasium access but without implementing physical-activity-related policies or practices. Cluster 1, which comprised schools predominantly located in rural areas, was termed “willing but unable”, while Cluster 2, which was primarily composed of urban schools, was dubbed “able but unwilling”. A minor segment within Cluster 1 lacked both infrastructure and policies, leading us to classify them as “unwilling and unable”. This differentiation underscores the specific domains into which public policy efforts can be most effectively channeled, mainly through infrastructural investments in schools which are “willing but unable” to promote physical activity or through the initiation of proactive programs in promoting a healthy lifestyle in schools that are “able but unwilling”.

School-based nutrition and physical activity interventions can significantly mitigate the obesogenic impact of the school environment, as highlighted by studies such as the Myheart Beat Study. This clustered, randomized control study showcased a reduction in overall dietary and carbohydrate intake when implementing school-wide subsidization for fruits and vegetables and healthy cooking preparation training for canteen and convenience shop operators [34,35]. While the efforts of the Myheart Beat study were channeled toward healthy eating, other notable intervention programs such as the Schools in Motion initiative, designed by the research and development team of the University of Tartu, targeted the improvement of physical activity in schools. Their endeavors documented the entire process from the pilot study stage to a nationally scaled program [36].

### Strengths and Limitations

Several limitations are evident in this research. Firstly, objective metrics such as BMI measurements or accelerometry, which were employed in other studies [30,31,32,33] to assess student weight status and measure sedentary or MVPA duration, were not utilized. Such measures would have provided a clearer, quantitative picture of weight balance disparities between urban and rural settings, thereby enriching the depth of the analysis.

Secondly, unlike the original study [23], an audit-based assessment of school facilities was not conducted. The reliance on administratively reported data without objective verification introduces the possibility of biases. Specifically, there was potential for recall bias in which the respondents might not accurately remember details about school practices, policies, or facilities. Social desirability bias may also play a role, with some schools potentially providing answers they deemed more favorable or socially acceptable rather than an accurate depiction of their actual practices and conditions. Despite achieving a response rate of 71.2%, the characteristics of the non-responding schools remain unknown, introducing a possible response bias.

Lastly, although the cross-sectional design of this research offers an important snapshot, it does not allow for insights into causal relationships or changes over time in school environments. Additionally, the sample was not randomly selected and was restricted to schools within Sibiu County.

Notwithstanding, the present methodology offers the advantage of being easily applicable and cost-effective. Moreover, it provides direction for interventions grounded in the existing literature, which has established correlations between positive changes in school policies, practices, and facilities, and the enhancement of physical activity and the promotion of healthy eating habits. This foundation ensures that the proposed changes have empirical backing, reinforcing their potential effectiveness. Moreover, the study’s innovative two-step cluster analysis stands out as a unique approach, offering a more nuanced understanding of school clusters and pinpointing precise areas in which interventions can be the most impactful. By merging cost efficiency with methodological ingenuity, this research maintains its practical and strategic utility in understanding and improving school environments despite the limitations encountered.

## 5. Conclusions

In summary, the adaptation and validation of the ISCOLE School and Environment questionnaire for Romanian schools yielded significant insights into the obesogenic impact of school environments. This study encompassed a comprehensive range of schools and highlighted notable disparities between rural and urban settings, underscoring the imperative need for targeted public policy interventions. Through a nuanced cluster analysis, this study identified specific school clusters, emphasizing areas in which investments or initiatives could be effectively channeled. Despite certain limitations, the adopted methodology stands out for its cost-effectiveness and practical applicability. By drawing on the established literature, the present research underscores the importance of fostering health-promoting school environments, aiming to enhance physical activity and advocate for healthy eating habits across all school levels. Assessing obesogenic school environments in Sibiu County provides a pilot model for broader applications due to its diverse school landscape and supportive local authorities. The findings emphasize the importance of optimizing school environments to foster healthier lifestyles, which could have long-term implications on children’s well-being, reducing potential health complications and burdens on the healthcare system in the future. Prioritizing changes based on empirical data could lead to more effective, tailored interventions that could substantially improve the quality of life of students and potentially serve as a model for other regions.

## Figures and Tables

**Table 1:** School characteristics.

Variable	Descriptive Parameter	Environment		*p*-Value
		Rural	Urban	
No. of students	Mean	276.9	644.4	<0.01
	StdDev	169.4	370.5	
	IQR	160	571	
	MIN	43	92	
	MAX	849	2000	
	95%CI	220.4–333.4	535.6–753.2	
No. of classes	Mean	17.46	28.91	<0.01
	StdDev	10.61	13.44	
	IQR	11	18	
	MIN	5	9	
	MAX	60	76	
	95%CI	13.9–21	25–32.9	
Average no. of students/class	Mean	15.8	21.41	<0.01
	StdDev	3.5	5.27	
	IQR	6	5.6	
	MIN	8.6	6.7	
	MAX	21.67	29.7	
	95%CI	14.7–177	19.9–233	
No. of teachers (full-time equivalents)	Mean	26.3	47.8	<0.01
	StdDev	14.7	21.3	
	IQR	18.5	30	
	MIN	8	17	
	MAX	83	130	
	95%CI	21.4–32.2	41.5–54	

StdDev—standard deviation; IQR—interquartile range; MIN—the minimum value in the sample; MAX—the maximum value in the sample; 95%CI—95% confidence interval for the mean.

**Table 2:** Hours of the curriculum per each school’s decision (CDS) dedicated to physical activity and healthy eating.

Hours of the CDS Dedicated to	Response	Environment		*p*-Value
		Rural (%)	Urban (%)	
Physical activity	No	24 (64.9%)	26 (55.3%)	0.686
	Yes (some classes)	3 (8.1%)	6 (12.8%)	
	Yes (all)	10 (27%)	15 (31.9%)	
Healthy eating	No	11 (29.7%)	16 (34%)	0.555
	Yes (some classes)	8 (21.6%)	6 (12.8%)	
	Yes (all)	18 (48.6%)	25 (53.2%)	

**Table 3:** Policies and practices regarding physical activity and healthy eating across environments.

Policies or Practices Concerning	Response	Environment		*p*-Value
		Rural (%)	Urban (%)	
Physical activity	No	6 (16.2%)	9 (19.1%)	0.781
	Yes	31 (83.8%)	38 (80.9%)	
Healthy eating	No	7 (18.9%)	9 (19.1%)	0.602
	Yes	30 (81.1%)	38 (80.9%)	

**Table 4:** Transportation provided to extracurricular activities.

Organized Transport	Environment		*p*-Value
	Rural (%)	Urban (%)	
No	2 (5.4%)	15 (31.9%)	<0.01
Yes, sometimes	7 (18.9%)	14 (29.8%)	
Yes, always	28 (75.7%)	18 (38.3%)	

**Table 5:** School facilities available for physical across environments.

Available Facility	Environment		*p*-Value
	Rural (%)	Urban (%)	
Gym	24 (64.9%)	43 (91.5%)	<0.01
Other large halls or spaces	16 (43.2%)	20 (42.6%)	0.949
Running track	7 (18.9%)	7 (14.9%)	0.623
Outdoor sports ground	31 (83.8%)	43 (91.5%)	0.324
Paved area	32 (86.5%)	38 (80.9%)	0.491
Secured lockers	10 (27%)	32 (68.1%)	<0.01
Showers	3 (8.1%)	15 (31.9%)	<0.01
Bicycle racks	9 (24.3%)	31 (66%)	<0.01
Lawn-covered area	21 (56.8%)	18 (38.3%)	0.092
Fixed-equipment playground	23 (62.2%)	21 (44.7%)	0.111
Art room	11 (29.7%)	7 (14.9%)	0.1
Music room	2 (5.4%)	4 (8.5%)	0.458

**Table 6:** School eating facilities across environments.

Available Facility	Environment		*p*-Value
	Rural (%)	Urban (%)	
National programs such as the “Milk and Breadstick” initiative	37 (100%)	36 (76.6%)	<0.01
Canteen/Cafeteria	2 (5.4%)	6 (12.8%)	0.225
In-school store	2 (5.4%)	5 (10.6%)	0.327
Store near the school	22 (59.5%)	31 (66%)	0.540
Fast-food restaurants	2 (5.4%)	13 (27.7%)	<0.01
Vending machines (drinks)	0 (0%)	6 (12.8%)	0.026
Vending machines (snacks)	0 (0%)	1 (2.1%)	0.560

**Table 7:** Healthy eating activities across environments.

Activity Implemented	Environment		*p*-Value
	Rural (%)	Urban (%)	
Written information	33 (89.2%)	41 (87.2%)	1
Field trips to local producers	17 (45.9%)	26 (55.3%)	0.510
Cultivating produce	15 (40.5%)	13 (27.7%)	0.249
Cooking classes	1 (2.7%)	5 (10.6%)	0.222
Informative activities (in the 12 months previous to enrollment)	30 (81.1%)	44 (93.6%)	0.078
Special events (in the 12 months previous to enrollment)	9 (24.3%)	12 (25.5%)	1

**Table 8:** Problems regarding school surroundings.

Problem	Problem Intensity	Environment		*p*-Value
		Rural (%)	Urban (%)	
Ethnic or religious tensions	Not a problem	21 (56.8%)	39 (83%)	0.06
	Minor	8 (21.6%)	5 (10.6%)	
	Moderate	3 (8.1%)	1 (2.1%)	
	Major	5 (13.5%)	2 (4.3%)	
Garbage	Not a problem	7 (18.9%)	24 (51.1%)	<0.01
	Minor	10 (27%)	12 (25.5%)	
	Moderate	9 (24.3%)	8 (17%)	
	Major	11(29.7%)	3 (6.4%)	
The sale of alcohol	Not a problem	17 (45.9%)	25 (53.2%)	0.464
	Minor	7 (18.9%)	8 (17%)	
	Moderate	3 (8.1%)	7 (14.9%)	
	Major	10 (27%)	7 (14.9%)	
Drug use	Not a problem	25 (67.6%)	32 (68.1%)	0.304
	Minor	5 (13.5%)	5 (10.6%)	
	Moderate	0 (0%)	4 (8.5%)	
	Major	7 (18.9%)	6 (12.8%)	
Gangs	Not a problem	21 (56.8%)	29 (61.7%)	0.469
	Minor	7 (18.9%)	9 (19.1%)	
	Moderate	5 (13.5%)	8 (17%)	
	Major	4 (10.8%)	1 (2.1%)	
Heavy road traffic	Not a problem	10 (27%)	9 (19.1%)	0.128
	Minor	15 (40.5%)	11 (23.4%)	
	Moderate	4 (10.8%)	12 (25.5%)	
	Major	8 (21.6%)	15 (31.9%)	
Abandoned buildings	Not a problem	26 (70.3%)	30 (63.8%)	0.290
	Minor	4 (10.8%)	12 (25.5%)	
	Moderate	4 (10.8%)	2 (4.3%)	
	Major	3 (8.1%)	3 (6.4%)	
Crime rate	Not a problem	26 (70.3%)	37 (78.7%)	0.392
	Minor	4 (10.8%)	7 (14.9%)	
	Moderate	2 (5.4%)	1 (2.1%)	
	Major	5 (13.5%)	2 (4.3%)	

**Table 9:** Two-step cluster analysis model (PA—physical activity).

Variable	Descriptive Parameter	Cluster 1	Cluster 2	Cluster 3	Cluster 4	*p*-Value	Predictor Importance
Count	-	14 (16.7%)	12 (14.3%)	20 (23.8%)	38 (45.2%)		
No. of teachers (full-time equivalents)	Mean	21	33.6	68.6	30.2	<0.01	1
	StdDev	11	15.9	17.8	8.3		
	IQR	14.6	30.8	18.8	9.5		
	MIN	8	15	50.6	14		
	MAX	47	58.9	130	49		
	95%CI	14.7–27.3	23.5–43.7	60.3–76.9	27.5–33		
No. of students	Mean	201.9	360.3	958.4	374.1	<0.01	0.88
	StdDev	155.6	283	323.1	138.3		
	IQR	141.8	289.3	255.3	214		
	MIN	43	92	594	125		
	MAX	670	973	2000	640		
	95%CI	112.1–291.7	180.4–540.1	807.2–1109.6	328.6–419.5		
PA policies or practices	Yes	11 (78.6%)	0 (0%)	20 (100%)	38 (100%)	<0.01	0.77
	No	3 (21.4%)	12 (100%)	0 (0%)	0 (0%)		
Access to a gymnasium	Yes	0 (0%)	12 (100%)	17 (85%)	38 (100%)	<0.01	0.77
	No	14 (100%)	0 (0%)	3 (15%)	0 (0%)		

StdDev—standard deviation; IQR—interquartile range; MIN—the minimum value in the sample; MAX—the maximum value in the sample; 95%CI—the 95% confidence interval for the mean.

**Table 10:** Cluster comparison regarding environment.

Environment	Cluster 1	Cluster 2	Cluster 3	Cluster 4	*p*-Value
Rural	11 (78.6%)	4 (33.3%)	2 (10%)	20 (52.6%)	<0.01
Urban	3 (21.4%)	8 (66.7%)	18 (90%)	18 (47.4%)	

## Data Availability

The data presented in this study are available upon reasonable request from the corresponding author.

## Supplementary Materials

The following supporting information can be downloaded at https://www.mdpi.com/article/10.3390/children10111746/s1, Scan S1: backward translation; Table S1: Original ISCOLE questions, forward and backward translations or adaptations.

## Appendix A

Examples of school-level exposures affecting body weight and Romanian legislation regarding the offer of meals during school for students and preschoolers.

**Table A1:** Examples of school-level exposures affecting body weight.

Influence Category	Exposure Type	Exposure	Potential Regulatory Factors
Nutrition	Beneficial	Offering a free meal during school [37].	State policies regarding offering meals during school, e.g., the pilot program “Hot Meals in Schools” implemented in Romania or the “milk and breadstick” program.
	Detrimental	The ease of access to competitive foods [38] or advertising thereof [39].	State policies regulating foods with high sugar content, e.g., the UK’s sugar tax [40]. State policies restricting food and beverages in school cafeterias, e.g., Brazil [41]. Restrictions on competitive food sales and advertisements extending to the areas around schools, e.g., India (50 m radius) [42].
Physical activity	Beneficial	Longer morning breaks, the provision of road safety features [43].	School and state policies
	Detrimental	Lack of access to facilities enabling physical activity [44,45,46].	Enhancing school playground facilities [47].

Romanian legislation regarding the offer of meals during school for students and preschoolers:

The pilot program of offering one hot meal during school.

Emergency Ordinance of the Government No. 72/2016 regarding the approval of the Pilot Program for providing food support to preschoolers and students from 50 state pre-university educational institutions, approved by Law No. 92/2017.

Emergency Ordinance of the Government No. 92/2017 regarding the approval of the Pilot Program for providing food support to preschoolers and students from 50 state pre-university educational institutions, approved with modifications by Law No. 107/2018.

Emergency Ordinance of the Government No. 97/2018 regarding the approval of the Pilot Program for providing food support to preschoolers and students from 50 state pre-university educational institutions, approved by Law No. 201/2019.

Emergency Ordinance of the Government No. 9/2020 regarding the approval of the Pilot Program for providing food support to preschoolers and students from 150 state pre-university educational institutions, approved by Law No. 26/2021.

Emergency Ordinance No. 91/2021 regarding the approval of the continuation of the Pilot Program for providing food support to preschoolers and students from 150 state pre-university educational institutions, approved with modifications and additions by Law No. 306/2021.

Ordinance No.105/2022 regarding the approval of the continuation of the Pilot Program for providing food support to preschoolers and students from 300 state pre-university educational institutions, with subsequent modifications.

The “Milk and Breadstick” program.

Government Ordinance No. 13/2017 regarding the approval of Romania’s participation in the European Union’s School Program, approved with additions by Law No. 55/2018, with subsequent amendments and completions.

## Appendix B

Questionnaire changelog.

A brief section for initial instructions was added.

An initial query regarding the name of the school for which the response was filled out was added.

Question no. 1 from the ISCOLE School Environment Questionnaire allowed for the selection of “other” as a response option. This was removed in the adapted questionnaire (Question 2). Instructions from the Sibiu County Board of Education specified that the respondents should be either the headmaster, the deputy headmaster, or part of the faculty of the school.

Question no. 4 from the ISCOLE School Environment Questionnaire (“What grades are taught at your school?”) was worded as “Please select the type of education offered by the school you are attending (select all corresponding answers):” with the options “Primary education-classes I–IV/Secondary Education—Classes V–VIII/High School Education-Classes IX–XIII”. This is due to the particular organization of the national education system whereby schools either teach an entire category of classes (i.e., primary, lower secondary, or higher secondary) as a whole or not at all.

Question no. 5 from the ISCOLE School Environment Questionnaire, (“How many days (excluding holidays) do your students attend school during the academic school year?”) was removed. The number of days on which school is attended during the academic school year, as well as the start and end of the academic school year and school holidays, are established yearly by the Ministry of Education and apply to all the schools that could potentially be included in the current study.

Two queries (Questions 6 and 7 of the adapted questionnaire) were incorporated into the survey to ascertain the allocation of hours for physical activity and the promotion of healthy eating habits within the school-decided curriculum or locally developed curriculum. This type of curriculum is a part of the national curriculum and is tailored by each school to meet the specific learning needs of its students. The CDS focuses on optional subjects and study domains which can be offered either nationally or by individual schools. The hours dedicated to CDS are predefined and mandatory to follow based on each education level and track.

For Questions 6 and 7 of the ISCOLE questionnaire, regarding the presence of policies and practices within schools, the option “N/A” was excluded as it provided no additional information in the specific setting of the current study.

Follow-up questions were added after the initial queries regarding the presence of school practices or policies regarding physical activity and healthy eating whereby the respondents were asked to elaborate and describe specific policies and practices adopted within their institution.

Questions targeted toward a specific grade (i.e., “grade 4” in the ISCOLE questionnaire) were removed. The reason for this decision was that the ISCOLE study was aimed at a much narrower age interval in the studied population (9–11 years), while the present study focused on a broader age range (and therefore more grades) in a more restricted geographical area.

The answer option “Academic/hobby clubs (e.g., chess, astronomy)” from Question 9 of the ISCOLE School Environment Questionnaire was divided into two possible options, one for academic clubs and one for hobby clubs.

The answers to Question 10 from the iscole questionnaire (“does your school offer late bus/transportation service to students who participate in extracurricular activities?”) were rephrased from yes/no to yes, always/yes, sometimes/no.

The possible answers to Question 11 from the ISCOLE questionnaire (“From the following list, please indicate which sports are offered in your interschool or intramural athletics programs available to students in grade 4”) were adapted to reflect local options practiced more commonly, i.e., “Baseball/Softball”, “Rugby”, “Ice Hockey”, “Lacrosse”, “Track & Field”, “Swimming”, “Skiing”, and “Ultimate Frisbee” were removed, and “Tennis” was added.

Questions 10 and 11 from the ISCOLE questionnaire had their order reversed.

A follow-up question was added after Question 11 of the ISCOLE questionnaire, “From the following list, please indicate which sports are offered in your interschool or intramural athletics programs available to students in grade 4” in order to obtain further details if the respondents selected the option “Other”.

Question no. 14 of the original ISCOLE School Environment Questionnaire (“How much class time is mandated by your [Province/Territory/State…] to be allotted to physical education (PE)/Daily physical Activity (DPA) for students in grade 4”) and Question 15 (“Compared to the class time allotted to physical education (PE)/Daily Physical Activity (DPA) for grade 4 as mandated by your [Province/Territory/State…], do students in grade 4 in your school receive on average”) were removed. This was because PE activity is regulated by the Ministry of Education according to the curricula specific to each education level and track. The only alternative method of spending time during classes engaged in physical education or daily physical activity is by allotting hours from the school-decided curriculum, which was addressed in the queries added before.

The option “don’t know” was removed for Questions 16–18, 20, and 21 of the original ISCOLE questionnaire.

For Question 16 of the original questionnaire, the answers were replaced with a five-item scale as follows: “Not at all/very little/little/much/very much”.

For Question 17 of the original questionnaire, the answer regarding the provision of crossing guards or car-free zones was removed as there is no applicable legal setting for restricting or directing traffic on behalf of school administrations in Romania.

For Question 18 of the original ISCOLE questionnaire (“Do the majority of students at your school have regular access to any of the following during school hours?”), the answers “l. Bicycle racks”, and “m. If yes, are the racks in a secure area to avoid theft?” were combined into a single option: “bicycle rack in an area that avoids theft”. In addition, the answer options were simplified into yes or no variants.

Question 19 of the original ISCOLE questionnaire was moved after Question 21.

The option “N/A” was removed from Question 20 of the original ISCOLE questionnaire as this could be deduced from Question 18 in the context of local regulations (i.e., if students did not have access during school hours to a certain facility, it implies that the school did not possess that facility).

Questions 22 (“Does your school provide any of the following to promote the sale of healthy food?”), 23 (“Does your school ensure that all students, regardless of ability to pay, have access to fruits and vegetables?”), and 26 (“During the past 12 months, have any of the following items been sold as part of fundraising for any school organization?”) of the original ISCOLE questionnaire were removed. Regarding Question 23, school administrations do not have the legal or administrative capacity to provide access to foods other than those permitted to be sold on school grounds and cannot influence their price. The foods sold within schools are strictly regulated by law in Romania (OMSP 1563/2008). As such, the food items mentioned in Question 26 cannot be sold on school premises. Options from Question 22 are rendered either impossible to select on the same premises (i.e., “Healthy food choices at a reasonable/subsidized price” and “Healthy eating cafeteria program (e.g., Eat Smart or independent program”) or redundant due to inquiries regarding healthy eating policies or practices throughout the rest of the questionnaire (i.e., “Healthy eating promotional materials (e.g., posters)” and “Daily healthy eating specials”.

The options “Field trips to farms/farmers’ markets” and “Field trips to the local grocery store” from Question 24 of the original ISCOLE questionnaire were replaced with “trips to local food producers” to more accurately reflect local practices.

For Question 25 of the original ISCOLE questionnaire, the options “Offered healthy food choices during breakfast program”, “Offered healthy food choices during lunch program”, “Offered healthy food choices in the snack bar/school shop(s)”, and “Stopped the sale of junk food” were removed due to similar reasons for which Questions 22 and 23 were removed. The option “Organized Nutrition Month activities” was changed to a more generic “Organized informative activities for Healthy Nutrition”, and the option “held junk food free days” was changed to “organized activities type” day without sweetened drinks”.

An additional option, the “sale of alcoholic beverages in the surroundings of the school”, was added to Question 27 of the original ISCOLE questionnaire.

## Appendix C

Practices regarding physical activity and healthy eating in Romanian schools—honorable mentions.

The following projects from our respondents stood out as noteworthy examples of innovative approaches to physical activity and healthy eating practices:

“Sport: Nature and Adventure”—Păuca Elementary School. This project is a collaboration between the Păuca Elementary School and the Sibiu County Council and Păuca City Hall which entail a series of activities in which children from the villages of the Păuca Commune are encouraged to practice sports, adhere to nutrition guidelines, spend more time outdoors, and protect nature. At the end of the competitions, “all children were declared winners” and received prizes and medals. The project was funded by the Sibiu County Council.

Participation in the “Urme pe play” marathon by the “Sava Popovici Barcianu” Elementary School of Rășinari. “Urme pe play” is a clever play on words in Romanian. While “urme pe plai” means “tracks on a plain,” the word “plai” is intentionally replaced with “play” to hint at the playful aspect of the event, emphasizing its fun and engaging nature. Students at this school were encouraged to participate in the marathon, which spans the rural area around the Cindrel and Lotru mountains in Sibiu County.

The “Yummy marathon” organized by the “Automecanica” Technical High School of Mediaș is a cook-off organized yearly by the school in which students are awarded prizes for their take on nutrition and focusing on healthy alternatives.
