# Supplementary material for: Assessing Obesogenic School Environments in Sibiu County, Romania: Adapting the ISCOLE School Environment Questionnaire
Source: Children (Basel). 2023 Oct 27;10(11):1746. doi: 10.3390/children10111746 (PMC10670591; doi:10.3390/children10111746)
Supplement: Supplementary file 1 [file children-10-01746-s001.zip › Scan S1.pdf]

Influence of the school environment on obesity in children

Please read each question carefully.
Mark each question with the answer that suits you best. To remember:
- This is not a test so there are no wrong answers.
- The data obtained are confidential.

A. Characteristics of the school

1. Please specify the name of the school where you work.
2. What is your position in this school?

   Principal / deputy principal / teacher

3. What is the approximate number of students at the school where you work?
4. What is the approximate number of teaching positions at the school where you work?
5. Please select the type of education offered by the school you are attending (select all corresponding answers):

   Primary education-classes I-IV / Secondary Education - Classes V-VIII / High School Education-Classes IX-XIII

6. Does your school allocate **physical activity** hours from CDS/CDL (curriculum at school decision/curriculum in local development)?

   Yes, in all classes / yes, but only at certain levels/majors / no

7. Does your school allocate CDS/CDL classes (curriculum at school decision/curriculum in local development) to promote **healthy eating**?

   Yes, in all classes / yes, but only at certain levels/majors / no

The undersigned, Matei Rodica, authorised interpret and translator for the foreign languages English-French-Spanish based on the authorisation no. 27195290 from 2010, issued by the Ministry of Justice from Romania, certify the accuracy of the translation made from Romanian into English that the text shown to me was translated in excerpt without omissions and that by the translation the content and the meaning of the document was not modified.

INTERPRET AUTORIZAT DE MINISTERUL JUSTITIEI ROMANIA CIF 27195290 MATEI RODICA Engleză, Franceză Spaniolă AUT. 29028/2010

B. School policies and practices on healthy eating and physical activity

In this section, the term "*policies*" refers to **regulations** imposed at the state level (for example, the "*Milk and Croissant*" program) or by school management or other authorities capable of influencing the school environment that have been formally **adopted**. The term '*practices*' refers to activities and initiatives that are allowed to school pupils and teachers **outside curricula or physical education classes** to promote a healthy lifestyle or to conduct events involving physical activity.

8. Are there any *physical* activity *policies* or **practices** in your school? For example:

Policies: allowing access to out-of-hours school facilities for physical activity

Practices: periodic mountain trips, hiking.

Yes, there are written policies / Yes, there are policies, but they are not yet written / Yes, there are practices
/ No policies or practices

9. If you answered yes to the previous question, please elaborate on the *policies* and/or *practices* implemented at the level of the school you work in for the promotion of physical activity.

Mention the policy or practice and provide a brief description. For example:

Policies to promote daily activity by allowing access to sports equipment during the break (ex. basketballs, tennis rackets, etc.)

Practice to promote daily activity through the annual Organization of a ski Cup.

10. Are there **healthy eating** *policies* or *practices* in your school?

For example: banning certain foods in school (**policies**), events centered on healthy cooking (**practice**) etc.

Yes, there are written policies / Yes, there are policies, but they are not yet written / Yes, there are practices
/ No policies or practices

11. If you answered YES to the previous question, please elaborate on the *policies* and/or *practices* implemented at the level of your school to promote healthy eating.

12. Is there a *committee* or other organized form at school level dealing with proposals on the development of policies and/or practices on physical activity and healthy eating?

For example, members of the student council responsible for these tasks, members of the parents ' committee or of the teaching staff grouped in a commission or other form of organization, etc.

Yes - both for physical activity and for healthy eating / Yes - only for physical activity / Yes-only for healthy eating / no

The undersigned, Matei Rodica, authorised interpret and translator for the foreign languages English-French-Spanish based on the authorisation no. 27195290 from 2010, issued by the Ministry of Justice from Romania, certify the accuracy of the translation made from Romanian into English that the text shown to me was translated in excerpt without omissions and that by the translation the content and the meaning of the document was not modified.

CIF 27195290 MATEI RODICA Engleză, Franceză Spaniolă AUT. 27195290/2010

C. Physical activity in school

13. Please estimate what percentage of the school's students participate in the following activities:

Inter-school sports competitions / School sports clubs/courses (including dance) / School clubs of training courses / School hobby clubs/courses (chess, modeling, etc.), School art clubs/courses (drawing, music, and theatre, photo, etc.);

Unavailable / <10% / 10-24% / 25-49% / 50% +

14. Please select which of the following sports is practiced **in an organized** manner **extracurricularily** in your school in the form of training and/or inter-school competitions.

Basketball / Volleyball / Football / Tennis / Gymnastics / Badminton / Other;

Extracurricular training in school / inter-school competitions / not applicable

15. If you answered the previous question, with "Other" please which one.

16. Does your school offer organized transportation to students participating in extracurricular activities?

Yes, always / yes, sometimes / no

17. How many **15-29 minute breaks** do students studying at your school have per day?

0 / 1 / 2 / 3 or more

18. How many breaks of **30 minutes or more** do students studying at your school have per day?

0 / 1 / 2 / 3 or more

19. As far as you know, how do you assess the truth value of the following statements?

We use physical activity as rewards / promote physical activity

in special events /

We integrate physical activity into other curricula outside of sports hours (e.g. in the form of outdoor classes) /

We use physical activity as punishment (e.g. Prohibition of leaving class on break, doing field laps, etc.);

Not at all / very little / little / much / very much

The undersigned, Matei Rodica, authorised interpret and translator for the foreign languages English-French-Spanish based on the authorisation no. 27195290 from 2010, issued by the Ministry of Justice from Romania, certify the accuracy of the translation made from Romanian into English that the text shown to me was translated in excerpt without omissions and that by the translation the content and the meaning of the document was not modified.

CIF 27195290
MATEI
RODICA
Engleză, Franceză
Spaniolă
[illegible]28/2010

20. As far as you know, does your school promote **active physical** transportation of students to school in any of the following ways?

Identification of safe routes for pedestrian or bicycle access to the school (e.g. marking by articles in the magazine of the school or on the website of the institution, posters etc.) /

Bicycle access on the property /

Allowing access with rollers, skateboards or scooters on the property /

Encouraging the wearing of bicycle helmets or other protective equipment for students using bicycles / rollers/scooters/etc. for transport to the school (including through posters displayed in the school, etc.) /

Organization of events such as "walking bus" (Organization of meeting points, similar to bus stops, to gather students to continue walking to the school) or "day of cycling to school";

Yes/No

D. School facilities

21. Which of the following facilities do your school's **students have access to during school hours** (including sports classes)?

Gym / other large halls or spaces that allow physical activity (dance hall, celebration hall, amphitheatre, etc.) / running track / Outdoor Sports Ground (football, basketball, etc.) / paved area for other physical activities (exclotron, jumping rope, etc.) / roller track / indoor pool / secure locker area usable during physical activity / showers available before and after physical activity / bicycle rack in an area that avoids theft / lawn covered area / playground with fixed equipment slide, swings, ping-pong table, etc.) / room for artistic activities (painting, sculpture, etc.) / music room;

Yes / No

22. Which of the following school facilities do your school's students have access to **outside of school hours**?

Gym / halls located inside the building / outdoor facilities (ex. basketball court, etc.) / sports equipment (ex. basketballs, etc.);

Yes/No

23. Does your school allow **out-of-class** access to organized groups for the use of physical activity facilities?

Yes / No

The undersigned, Matei Rodica, authorised interpret and translator for the foreign languages English-French-Spanish based on the authorisation no. 27195290 from 2010, issued by the Ministry of Justice from Romania, certify the accuracy of the translation made from Romanian into English that the text shown to me was translated in excerpt without omissions and that by the translation the content and the meaning of the document was not modified.

CIF 27195290 MATEI RODICA Engleză, Franceză Spaniolă Aut. 29028/2010

24. Which of the following facilities do your school's students have access to for food procurement?

Canteen / shop inside the school / shops near the school / fast food restaurants near the school / appliances / vending machines with sweets or potato chips or other snacks / appliances/ vending machines with drinks / Program type "milk and croissant" - only for the classes to which it applies;

Yes/No

E. Healthy Eating

25. Are any of the following available in your school? Mark all the answers that correspond.

Cooking classes / vegetable and fruit growing activities / trips to local food producers / written information on healthy eating

26. During the **last 12 months**, has your school organized any of the following activities?

Offered healthy food variants at the cafeteria / offered healthy food variants at the store of the school / offered healthy food variants at the food or beverage machines/ vending machines / organized informative activities for Healthy Nutrition / organized activities type "day without sweetened drinks"

Not applicable / Yes / No

F. School Surroundings

27. How much do you appreciate the following issues related to the area where the school is located to be a problem?

Tensions based on ethnic, religious or regional differences / garbage dumped in the surroundings of the school / sale of alcoholic beverages in the surroundings of the school / use or transaction of drugs in the surroundings of the school / gangs / neighborhood gangs / heavy road traffic / abandoned or damaged buildings / crime rate in the neighborhood

Major problem / moderate problem / minor problem / not a problem

The undersigned, Matei Rodica, authorised interpret and translator for the foreign languages English-French-Spanish based on the authorisation no. 27195290 from 2010, issued by the Ministry of Justice from Romania, certify the accuracy of the translation made from Romanian into English that the text shown to me was translated in excerpt without omissions and that by the translation the content and the meaning of the document was not modified.

INTERPRET AUTORIZAT MINISTERUL JUSTIȚIEI
CIF 27195290
MATEI
RODICA
Engleză, Franceză
Spaniolă
AUT. 29028/2010

Influence of the school environment on obesity in children

Please read each question carefully.
Mark each question with the answer that suits you best. To remember:
- This is not a test so there are no wrong answers.
- The data obtained are confidential.

A. Characteristics of the school

1. Please specify the name of the school where you work.
2. What is your position in this school?

   Principal / deputy principal / teacher

3. What is the approximate number of students at the school where you work?
4. What is the approximate number of teaching positions at the school where you work?
5. Please select the type of education offered by the school you are attending (select all corresponding answers):

   Primary education-classes I-IV / Secondary Education - Classes V-VIII / High School Education-Classes IX-XIII

6. Does your school allocate **physical activity** hours from CDS/CDL (curriculum at school decision/curriculum in local development)?

   Yes, in all classes / yes, but only at certain levels/majors / no

7. Does your school allocate CDS/CDL classes (curriculum at school decision/curriculum in local development) to promote **healthy eating**?

   Yes, in all classes / yes, but only at certain levels/majors / no

The undersigned, Muntean Antonia Cecilia, authorised interpret and translator for the foreign languages English-French based on the authorisation no. 26299 from 05.08.2009, issued by the Ministry of Justice from Romania, certify the accuracy of the translation made from Romanian into English that the text shown to me was translated in excerpt without omissions and that by the translation the content and the meaning of the document was not modified.

MUNTEAN ANTONIA-CECILIA
Aut. nr. 26299
Limba Engleză, Franceză
ROMANIA

B. School policies and practices on healthy eating and physical activity

In this section, the term "*policies*" refers to **regulations** imposed at the state level (for example, the "*Milk and Croissant*" program) or by school management or other authorities capable of influencing the school environment that have been formally **adopted**. The term '*practices*' refers to activities and initiatives that are allowed to school pupils and teachers **outside curricula or physical education classes** to promote a healthy lifestyle or to conduct events involving physical activity.

8. Are there any *physical* activity *policies* or **practices** in your school? For example:

Policies: allowing access to out-of-hours school facilities for physical activity

Practices: periodic mountain trips, hiking.

Yes, there are written policies / Yes, there are policies, but they are not yet written / Yes, there are practices
/ No policies or practices

9. If you answered yes to the previous question, please elaborate on the *policies* and/or *practices* implemented at the level of the school you work in for the promotion of physical activity.

Mention the policy or practice and provide a brief description. For example:

Policies to promote daily activity by allowing access to sports equipment during the break (ex. basketballs, tennis rackets, etc.)

Practice to promote daily activity through the annual Organization of a ski Cup.

10. Are there **healthy eating** *policies* or *practices* in your school?

For example: banning certain foods in school (**policies**), events centered on healthy cooking (**practice**) etc.

Yes, there are written policies / Yes, there are policies, but they are not yet written / Yes, there are practices
/ No policies or practices

11. If you answered YES to the previous question, please elaborate on the *policies* and/or *practices* implemented at the level of your school to promote healthy eating.

12. Is there a *committee* or other organized form at school level dealing with proposals on the development of policies and/or practices on physical activity and healthy eating?

For example, members of the student council responsible for these tasks, members of the parents ' committee or of the teaching staff grouped in a commission or other form of organization, etc.

Yes - both for physical activity and for healthy eating / Yes - only for physical activity / Yes-only for healthy eating / no

The undersigned, Muntean Antonia Cecilia, authorised interpret and translator for the foreign languages English-French based on the authorisation no. 26299 from 05.08.2009, issued by the Ministry of Justice from Romania, certify the accuracy of the translation made from Romanian into English that the text shown to me was translated in excerpt without omissions and that by the translation the content and the meaning of the document was not modified.

MUNTEAN ANTONIA CECILIA Aut. nr. 26299 Limba Engleză, Franceză ...-ROMANIA

C. Physical activity in school

13. Please estimate what percentage of the school's students participate in the following activities:

Inter-school sports competitions / School sports clubs/courses (including dance) / School clubs of training courses / School hobby clubs/courses (chess, modeling, etc.), School art clubs/courses (drawing, music, and theatre, photo, etc.);

Unavailable / <10% / 10-24% / 25-49% / 50% +

14. Please select which of the following sports is practiced **in an organized** manner **extracurricularily** in your school in the form of training and/or inter-school competitions.

Basketball / Volleyball / Football / Tennis / Gymnastics / Badminton / Other;

Extracurricular training in school / inter-school competitions / not applicable

15. If you answered the previous question, with "Other" please which one.

16. Does your school offer organized transportation to students participating in extracurricular activities?

Yes, always / yes, sometimes / no

17. How many **15-29 minute breaks** do students studying at your school have per day?

0 / 1 / 2 / 3 or more

18. How many breaks of **30 minutes or more** do students studying at your school have per day?

0 / 1 / 2 / 3 or more

19. As far as you know, how do you assess the truth value of the following statements?

We use physical activity as rewards / promote physical activity

in special events /

We integrate physical activity into other curricula outside of sports hours (e.g. in the form of outdoor classes) /

We use physical activity as punishment (e.g. Prohibition of leaving class on break, doing field laps, etc.);

Not at all / very little / little / much / very much

The undersigned, Muntean Antonia Cecilia, authorised interpret and translator for the foreign languages English-French based on the authorisation no. 26299 from 05.08.2009, issued by the Ministry of Justice from Romania, certify the accuracy of the translation made from Romanian into English that the text shown to me was translated in excerpt without omissions and that by the translation the content and the meaning of the document was not modified.

MUNTEAN ANTONIA-CECILIA Aut. nr. 26299 Limba Engleză, Franceză SIBIU - ROMANIA

20. As far as you know, does your school promote **active physical** transportation of students to school in any of the following ways?

Identification of safe routes for pedestrian or bicycle access to the school (e.g. marking by articles in the magazine of the school or on the website of the institution, posters etc.) /

Bicycle access on the property /

Allowing access with rollers, skateboards or scooters on the property /

Encouraging the wearing of bicycle helmets or other protective equipment for students using bicycles / rollers/scooters/etc. for transport to the school (including through posters displayed in the school, etc.) /

Organization of events such as "walking bus" (Organization of meeting points, similar to bus stops, to gather students to continue walking to the school) or "day of cycling to school";

Yes/No

D. School facilities

21. Which of the following facilities do your school's **students have access to during school hours** (including sports classes)?

Gym / other large halls or spaces that allow physical activity (dance hall, celebration hall, amphitheatre, etc.) / running track / Outdoor Sports Ground (football, basketball, etc.) / paved area for other physical activities (exclotron, jumping rope, etc.) / roller track / indoor pool / secure locker area usable during physical activity / showers available before and after physical activity / bicycle rack in an area that avoids theft / lawn covered area / playground with fixed equipment slide, swings, ping-pong table, etc.) / room for artistic activities (painting, sculpture, etc.) / music room;

Yes / No

22. Which of the following school facilities do your school's students have access to **outside of school hours**?

Gym / halls located inside the building / outdoor facilities (ex. basketball court, etc.) / sports equipment (ex. basketballs, etc.);

Yes/No

23. Does your school allow **out-of-class** access to organized groups for the use of physical activity facilities?

Yes / No

The undersigned, Muntean Antonia Cecilia, authorised interpret and translator for the foreign languages English-French based on the authorisation no. 26299 from 05.08.2009, issued by the Ministry of Justice from Romania, certify the accuracy of the translation made from Romanian into English that the text shown to me was translated in excerpt without omissions and that by the translation the content and the meaning of the document was not modified.

ANTONIA-CECILIA
Aut. nr. 262[illegible]
Limba Engleză, Franceză
[illegible]-ROMANIA

24. Which of the following facilities do your school's students have access to for food procurement?

Canteen / shop inside the school / shops near the school / fast food restaurants near the school / appliances / vending machines with sweets or potato chips or other snacks / appliances/ vending machines with drinks / Program type "milk and croissant" - only for the classes to which it applies;

Yes/No

E. Healthy Eating

25. Are any of the following available in your school? Mark all the answers that correspond.

Cooking classes / vegetable and fruit growing activities / trips to local food producers / written information on healthy eating

26. During the **last 12 months**, has your school organized any of the following activities?

Offered healthy food variants at the cafeteria / offered healthy food variants at the store of the school / offered healthy food variants at the food or beverage machines/ vending machines / organized informative activities for Healthy Nutrition / organized activities type "day without sweetened drinks"

Not applicable / Yes / No

F. School Surroundings

27. How much do you appreciate the following issues related to the area where the school is located to be a problem?

Tensions based on ethnic, religious or regional differences / garbage dumped in the surroundings of the school / sale of alcoholic beverages in the surroundings of the school / use or transaction of drugs in the surroundings of the school / gangs / neighborhood gangs / heavy road traffic / abandoned or damaged buildings / crime rate in the neighborhood

Major problem / moderate problem / minor problem / not a problem

The undersigned, Muntean Antonia Cecilia, authorised interpret and translator for the foreign languages English-French based on the authorisation no. 26299 from 05.08.2009, issued by the Ministry of Justice from Romania, certify the accuracy of the translation made from Romanian into English that the text shown to me was translated in excerpt without omissions and that by the translation the content and the meaning of the document was not modified.

ANTONIA-CECILIA
Aut. nr. 26299
Limba Engleză, Franceză
[illegible]-ROMÂNIA
