# Supplementary material for: Assessing Obesogenic School Environments in Sibiu County, Romania: Adapting the ISCOLE School Environment Questionnaire
Source: Children (Basel). 2023 Oct 27;10(11):1746. doi: 10.3390/children10111746 (PMC10670591; doi:10.3390/children10111746)
Supplement: Supplementary file 1 [file children-10-01746-s001.zip › Table S1.pdf]

Table S1 - *Original ISCOLE questions, forward and backward translations or adaptations*

	**Question, section title or description**
Original	**ISCOLE SCHOOL ENVIRONMENT QUESTIONNAIRE**
Adapted	**Influența mediului școlar asupra obezității la copii**
Backward	**Influence of the school environment on obesity in children**
Original	*(none)*
Adapted	Vă rugăm să citiți fi ecare întrebare cu atenție.<br>Marcați la fiecare întrebare răspunsul care se potrivește cel mai bine.<br>De ținut minte:<br>- Acesta nu este un test așa că nu există răspunsuri greșite.<br>- Datele obținute au caracter confi dențial.
Backward	Please read each question carefully.<br>Mark each question with the answer that suits you best. Remember:<br>- This is not a test, so there are no wrong answers.<br>- The data obtained are confidential.
Original	**A. SCHOOL CHARACTERISTICS**
Forward	**A. Caracteristicile școlii**
Backward	**A. Characteristics of the school**
Original	*(none)*
Adapted	1. Vă rugăm specificați cum se numește școala în care vă desfășurați activitatea profesională.
Backward	1. Please specify the name of the school where you work.
Original	**1. What is your position at this school?** ☐ Principal ☐ Vice Principal ☐ Teacher ☐ Other:____________
Forward	2. Care este funcția dumneavoastră în această școală?<br>*Director / Director adjunct / Profesor*
Backward	2. What is your position in this school?<br>*Principal / assistant principal / teacher*
Original	**2. What is the total number of students in your school?** (Please estimate) _____ students
Forward	3. Care este numărul aproximativ de elevi care învață la școala în care activați?
Backward	3. What is the approximate number of students at the school where you work?
Original	**3. What is the total number of teachers (full time equivalents) in your school?** (Please estimate) _____ teachers
Forward	4. Care este numărul aproximativ de posturi didactice la școala în care activați?
Backward	4. What is the approximate number of teachers at the school where you work?
Original	**4. What grades are taught at your school?** _____ to _____
Forward	5. Vă rugăm selectați tipul de învățământ oferit de școala la care activați (selectați toate răspunsurile care corespund):<br>*Învațământ primar - Clasele I-IV / Învățământ gimnazial - Clasele V-VIII / Învățământ liceal - Clasele IX-XIII*
Backward	5. Please select the type of education offered by the school you are attending (select all corresponding answers):<br>*Primary education-classes I-IV / Secondary Education - Classes V-VIII / High School Education-Classes IX-XIII*
Original	**5. How many days (excluding holidays) do your students attend school during the academic school year?** _______
Adapted	*(removed)*
Original	*(none)*
Adapted	6. Școala dumneavoastră alocă dintre orele CDȘ/CDL (curriculum la decizia şcolii/curriculum în dezvoltare locală) ore destinate **activității fizice**?<br>*Da, la toate clasele / Da, dar numai la anumite niveluri/specializări / Nu*
Backward	6. Does your school allocate **physical activity** hours from CDS/CDL (curriculum at school decision/curriculum in local development)?

	Yes, in all classes / yes, but only at certain levels/majors / No
Original	*(none)*
Adapted	7. Școala dumneavoastră alocă dintre orele CDȘ/CDL (curriculum la decizia şcolii/curriculum în dezvoltare locală) ore destinate promovării **alimentației sănătoase**?<br>*Da, la toate clasele / Da, dar numai la anumite niveluri/specializări / Nu*
Backward	7. Does your school allocate CDS/CDL classes (curriculum at school decision/curriculum in local development) to promote **healthy eating**?<br>*Yes, in all classes / yes, but only at certain levels/majors / No*
Original	**B. POLICIES AND PRACTICES**
Forward	**B. Politicile și practicile școlii privind alimentația sănătoasă și activitatea fizică**
Backward	**B. School policies and practices on healthy eating and physical activity**
Original	For the following section, "policies" refers to any mandates issued by the state, the local school board, or any other agency, including policies developed by your school or (district/diocese), that affects your school environment and that have been officially adopted by your school or district. This section also asks about practices (what your students and staff are allowed to do on a regular basis) that you might follow to promote the health and well-being of students.
Forward	În această secțiune termenul de *„politici”* se referă la **reglementări** impuse la nivel de stat (spre exemplu programul *„Laptele și cornul”*) sau de către conducerea școlii ori alte autorități capabile de a influența mediul din cadrul școlii care au fost adoptate în mod **oficial**. Termenul de *„practici”* se referă la activități și inițiative care sunt permise elevilor și profesorilor școlii **în afara curiculei sau a orelor de educație fizică** pentru promovarea unui stil de viață sănătos sau pentru desfășurarea de evenimente care implică activitatea fizică.
Backward	In this section, the term "*policies*" refers to **regulations** imposed at state level (for example, the "*Milk and Breadstick"* program) or by school management or other authorities capable of influencing the school environment that have been formally **adopted**. The term '*practices*' refers to activities and initiatives that are allowed to school students and teachers **outside curricula or physical education classes** to promote a healthy lifestyle or to conduct events involving physical activity.
Original	**6. Does your school have written policies or practices concerning physical activity?**<br>☐ **Yes**, existing written policies<br>☐ **Yes**, written policies still under development<br>☐ **Yes,** practices<br>☐ No<br>☐ N/A
Forward	8. Există în școala dumneavoastră *politici* sau *practici* privind **activitatea fizică**? De exemplu:<br>**Politică**: permiterea accesului la facilitățile școlii în afara programului în vederea activității fizice<br>**Practici**: excursii la munte periodice, drumeții.<br>*Da, există politici scrise / Da, există politici, dar nu sunt încă scrise / Da, există practici / Nu există politici sau practici*
Backward	8. Are there any *physical* activity *policies* or **practices** in your school? For example:<br>**Policies**: Policies allowing after-hours access to school facilities for physical activities.

	**Practices**: periodic mountain trips, hiking trips.<br>*Yes, there are written policies / Yes, there are policies, but they are not yet written / Yes, there are practices/ No policies or practices*
Original	*(none)*
Adapted	9. Dacă ați răspuns DA la întrebarea precedentă, vă rugăm elaborați asupra *politicilor* și/sau *practicilor* implementate la nivelul școlii în care activați pentru promovarea activității fizice.<br>Menționați politica sau practica și oferiți o scurtă descriere. De exemplu:<br>**Politică** de promovare a activității fizice prin permiterea accesului la echipament sportiv pe perioada pauzei (ex. mingi de baschet, rachete de tenis etc.)<br>**Practică** de promovare a activității fizice prin organizare anuală a unei cupe de schi.
Backward	*9. If you answered YES to the previous question, please elaborate on the policies and/or practices implemented at the level of the school you work in for the promotion of physical activity.*<br>*Mention the policy or practice* and provide a brief description. For example:<br>**Politics** to promote daily activity by allowing access to sports equipment during break (ex. basketballs, tennis rackets, etc.)<br>**Practice** to promote physical activity by organizing a yearly ski competition.
Original	**7. Does your school have written policies or practices concerning healthy eating?**<br>☐ **Yes**, existing written policies<br>☐ **Yes**, written policies still under development<br>☐ **Yes,** practices<br>☐ No<br>☐ N/A
Forward	10. Există în școala dumneavoastră *politici* sau *practici* privind **alimentația sănătoasă**?<br>De exemplu: interzicerea anumitor alimente în școală (**pollitică**), evenimente centrate pe gătitul în mod sănătos (**practică**) etc.<br>*Da, există politici scrise / Da, există politici, dar nu sunt încă scrise / Da, există practici / Nu există politici sau practici*
Backward	10. Are there **healthy eating** *policies* or *practices* in your school?<br>For example: banning certain foods in school (**politics**), events centered on healthy cooking (**practice**) etc.<br>*Yes, there are written policies / Yes, there are policies, but they are not yet written / Yes, there are practices / No policies or practices*
Original	*(none)*
Adapted	11. Dacă ați răspuns DA la întrebarea precedentă, vă rugăm elaborați asupra *politicilor* și/sau *practicilor* implementate la nivelul școlii în care activați pentru promovarea alimentației sănătoase.
Backward	11. If you answered YES to the previous question, please elaborate on the *policies* and/or *practices* implemented at the level of your school to promote healthy eating.

Original	**8. Does your school have a committee that oversees or offers guidance on the development of policies and practices concerning physical activity and healthy eating at your school (e.g., health action team, school health or wellness council)?**<br>☐ **Yes**, both physical activity and healthy eating<br>☐ **Yes**, physical activity only<br>☐ **Yes,** healthy eating only<br>☐ No
Forward	12. Există la nivelul școlii o *comisie* sau altă formă organizată care se ocupă cu propuneri privind dezvoltarea de politici și/sau practici privind activitatea fizică și alimentația sănătoasă?<br>De exemplu, membri din consiliul elevilor responsabili cu aceste sarcini, membri din comitetul părinților sau din corpul profesoral grupați într-o comisie sau altă formă de organizare etc.<br>*Da - atât pentru activitate fizică, cât și pentru alimentație sănătoasă / Da - doar pentru activitate fizică / Da - doar pentru alimentație sănătoasă / Nu*
Backward	12. Is there a *committee* or other organization at the school level in charge of proposals on the development of policies and/or practices on physical activity and healthy eating?<br>For example, members of the student council responsible for these tasks, members of the parents ' committee or of the faculty grouped in a committee or other form of organization, etc.<br>*Yes - both for physical activity and for healthy eating / Yes - only for physical activity / Yes - only for healthy eating / No*
Original	**C. PHYSICAL ACTIVITY**
Forward	**C. Activitatea fizică din școală**
Backward	**C. Physical activity in school**
Original	**9. What percent of students participate in the following extracurricular activities offered by your school?** (Please estimate)<br>Columns: *Not available* / *Less than 10%* / *10-24%* / *25-49%* / *50%+*<br>a. Varsity/interschool athletics ☐ ☐ ☐ ☐ ☐<br>b. Intramural athletics or physical activity clubs (including dance) ☐ ☐ ☐ ☐ ☐<br>c. Academic/hobby clubs (e.g., chess, astronomy) ☐ ☐ ☐ ☐ ☐<br>d. Arts-based clubs (e.g., drama, music, photography) ☐ ☐ ☐ ☐ ☐
Forward	13. Vă rugăm estimați ce procent de elevi ai școlii participă la următoarele activități:<br>*Campionate sportive interșcolare / Cluburi/cercuri sportive în școală (inclusiv dans) / Cercuri de pregătire pe discipline în școală / Cluburi/cercuri pentru hobby-uri în școală (șah, modelism, etc.) / Cluburi/cercuri artistice în școală (desen, muzică, teatru, fotografie, etc.);*<br>*Indisponibil / <10% / 10-24% / 25-49% / 50% +*
Backward	13. Please estimate what percentage of the school's students participate in the following activities:<br>*Inter-school sports competitions / School sports clubs/courses (including dance) / Academic clubs / School hobby clubs/courses (chess, modeling, etc.), School art clubs/courses (drawing, music, theatre, photo, etc.);*

<table>
<tr><td></td><td>Unavailable / <10% / 10-24% / 25-49% / 50%+</td></tr>
<tr><td>Original</td><td>11. From the following list, please indicate which sports are offered in your interschool or intramural athletics programs available to students in grade 4:
☐ a. Not applicable, school does not offer interschool or intramural athletics to students in grade 4
<table>
<tr><th></th><th>Varsity/ Interschool</th><th>Intramural</th><th></th><th>Varsity/ Interschool</th><th>Intramural</th></tr>
<tr><td>b. Basketball</td><td>☐</td><td>☐</td><td>j. Gymnastics</td><td>☐</td><td>☐</td></tr>
<tr><td>c. Volleyball</td><td>☐</td><td>☐</td><td>k. Wrestling</td><td>☐</td><td>☐</td></tr>
<tr><td>d. Soccer</td><td>☐</td><td>☐</td><td>l. Track & Field</td><td>☐</td><td>☐</td></tr>
<tr><td>e. Football</td><td>☐</td><td>☐</td><td>m. Badminton</td><td>☐</td><td>☐</td></tr>
<tr><td>f. Baseball/softball</td><td>☐</td><td>☐</td><td>n. Swimming</td><td>☐</td><td>☐</td></tr>
<tr><td>g. Rugby</td><td>☐</td><td>☐</td><td>o. Skiing</td><td>☐</td><td>☐</td></tr>
<tr><td>h. Ice Hockey</td><td>☐</td><td>☐</td><td>p. Ultimate Frisbee</td><td>☐</td><td>☐</td></tr>
<tr><td>i. Lacrosse</td><td>☐</td><td>☐</td><td>q. Other</td><td>☐</td><td>☐</td></tr>
</table></td></tr>
<tr><td>Forward</td><td>14. Vă rugăm selectați care dintre următoarele sporturi se practică organizat în mod extracuricular în școala dumneavoastră sub formă de antrenamente și/sau în competiții interșcolare.
Baschet / Volei / Fotbal / Tenis / Gimnastică / Badminton / Altele;
Antrenamente extracuriculare în școală / Competiții interșcolare / Nu se aplică</td></tr>
<tr><td>Backward</td><td>14. Please select which of the following sports is practiced in an organized manner as an extracurricular activity in your school in the form of training and/or inter-school competitions.
Basketball / Volleyball / Football / Tennis / Gymnastics / Badminton / Other;
Extracurricular training in school / inter-school competitions / not applicable</td></tr>
<tr><td>Original</td><td>(none)</td></tr>
<tr><td>Adapted</td><td>15. Dacă ați răspuns Altele la întrebarea precedentă, vă rugăm precizați care anume.</td></tr>
<tr><td>Backward</td><td>15. If you answered “Other” in the previous question, please specify which one.</td></tr>
<tr><td>Original</td><td>10. Does your school offer late bus/transportation service to students who participate in extra-curricular activities?
Yes ☐ No ☐</td></tr>
<tr><td>Forward</td><td>16. Școala dumneavoastră oferă transport organizat elevilor care participă la activități extracuriculare?
Da, întotdeauna / Da, uneori / Nu</td></tr>
<tr><td>Backward</td><td>16. Does your school offer transportation to students participating in extracurricular activities?
Yes, always / yes, sometimes / no</td></tr>
<tr><td>Original</td><td>For the following questions, please consider students in grade 4 when answering.</td></tr>
<tr><td>Adapted</td><td>(Removed)</td></tr>
<tr><td>Original</td><td>12. How many breaks of 15 to 29 minutes do students in grade 4 have in a day?
☐ zero ☐ 1 ☐ 2 ☐ 3 or more</td></tr>
</table>

Forward	17. Câte pauze de **15-29 de minute** au elevii care învață la scoala dumneavoastră pe zi?<br>*0 / 1 / 2 / 3 sau mai multe*
Backward	17. How many **15-29 minute** breaks do students at your school have per day?<br>*0 / 1 / 2 / 3 or more*
Original	**13. How many breaks of 30 minutes or more do students in grade 4 have in a day?**<br>☐ zero ☐ 1 ☐ 2 ☐ 3 or more
Forward	18. Câte pauze de **30 de minute sau mai mult** au elevii care învață la scoala dumneavoastră pe zi?<br>*0 / 1 / 2 / 3 sau mai multe*
Backward	18. How many breaks of **30 minutes or more** do students at your school have per day?<br>*0 / 1 / 2 / 3 or more*
Original	**14. How much class time is mandated by your [Province/Territory/State…] to be allotted to physical education (PE)/Daily Physical Activity (DPA) for students in grade 4?**<br>______ **minutes per** *[check the box indicating the time unit]* **week** ☐ **day** ☐<br>☐ No specific amount is mandated
Adapted	*(Removed)*
Original	**15. Compared to the class time allotted to physical education (PE)/Daily Physical Activity (DPA) for grade 4 as mandated by your [Province/Territory/State…], do students in grade 4 in your school receive on average:**<br>☐ Less than the mandated amount<br>☐ Approximately the mandated amount<br>☐ More than the mandated amount<br>☐ No specific amount is mandated
Adapted	*(Removed)*
Original	**16. To the best of your knowledge, how well do each of the following statements characterize your school?**<br>Columns: *A lot* / *Some* / *Very little* / *Not at all* / *Don't know*<br>a. We use physical activity as a reward ☐ ☐ ☐ ☐ ☐<br>b. We promote physical activity during or as part of special events ☐ ☐ ☐ ☐ ☐<br>c. We integrate physical activity into other curriculum areas ☐ ☐ ☐ ☐ ☐<br>d. We use physical activity as a punishment for bad behavior (e.g., withholding recess, administering push-ups or laps). ☐ ☐ ☐ ☐ ☐
Forward	19. Din câte știți, cum apreciați valoarea de adevăr a următoarelor afirmații?<br>*Utilizăm activitatea fizică sub formă de recompense /*<br>*Promovăm activitatea zică în cadrul unor evenimente speciale /*<br>*Integrăm activitatea fizică în cadrul altor curicule în afara orelor de sport (de exemplu sub forma orelor în aer liber) /*<br>*Utilizăm activitatea fizică sub formă de pedeapsă (de exemplu interzicerea părăsirii clasei în pauză, efectuarea de ture de teren etc.);*<br>*Deloc / Foarte puțin / Puțin / Mult / Foarte mult*
Backward	19. As far as you know, how do you assess the truth value of the following statements?

<table>
<tr><td></td><td>We use physical activity as reward/
We promote physical activity during special events /
We integrate physical activity into other curricula outside of sports hours (e.g. in the form of outdoor classes) /
We use physical activity as punishment (e.g. Prohibition of leaving class on break, doing field laps, etc.);
Not at all / very little / little / much / very much</td></tr>
<tr><td>Original</td><td>17. Does your school promote active transportation to and from school in any of the following ways?
<table>
<tr><th></th><th>Yes</th><th>No</th><th>Don't know</th></tr>
<tr><td>a. Identify safe routes to use for walking and cycling to and from school (e.g., with signs, in newsletters, etc.)</td><td>☐</td><td>☐</td><td>☐</td></tr>
<tr><td>b. Provide crossing guards at intersections to encourage safe walk-to-school routes</td><td>☐</td><td>☐</td><td>☐</td></tr>
<tr><td>c. Designate a 'car free zone' to provide safe walking areas around the school</td><td>☐</td><td>☐</td><td>☐</td></tr>
<tr><td>d. Allow students to bring bicycles on school property</td><td>☐</td><td>☐</td><td>☐</td></tr>
<tr><td>e. Allow students to bring small wheel vehicles (e.g., rollerblades, scooters, skateboards) on school property</td><td>☐</td><td>☐</td><td>☐</td></tr>
<tr><td>f. Encourage the use of helmets and safety gear for those who use bicycles and small wheel vehicles to get to school</td><td>☐</td><td>☐</td><td>☐</td></tr>
<tr><td>g. Organize occasional 'walk to school days' , walking clubs, or programs like 'walking school buses' (where parents or older students walk around the neighborhood and pick up walkers at designated points)</td><td>☐</td><td>☐</td><td>☐</td></tr>
</table></td></tr>
<tr><td>Forward</td><td>20. Din câte știți, promovează școala dumneavoastră transportul activ fizic al elevilor către școală în vreunul din următoarele moduri?
Identificarea rutelor sigure pentru accesul pedestru sau pe bicicletă către școală (ex: marcare prin articole în revista școlii sau pe site-ul instituției, afișe postate pe rute etc.) /
Permiterea accesului cu biciclete pe proprietatea școlii /
Permiterea accesului cu role, skate-boarduri sau trotinete pe proprietatea școlii /
Încurajarea purtării căștilor sau a altor echipamente de protecție pentru elevii care utilizează bicicletă/role/trotinetă/etc. pentru transportul către școală (inclusiv prin postere afișate în școală etc.) /
Organizare de evenimente de tipul „autobuzul pedestru” (organizare de puncte de întâlnire, similar unor stații de autobuz, pentru a aduna elevi care să meargă în continuare pe jos către școală) sau „ziua mersului cu bicicleta la școală”;
Da/Nu</td></tr>
<tr><td>Backward</td><td>20. As far as you know, does your school promote active physical transportation of students to school in any of the following ways?
Identification of safe routes for pedestrian or bicycle access to the school (e.g. marking by articles in the school magazine or on the institution's website, posters etc.) /
Bicycle access on school property /
Allowing access with rollers, skateboards, or scooters on schoolproperty /
Encouraging the wearing of bicycle helmets or other protective equipment for students using</td></tr>
</table>

<table>
<tr><td></td><td>bicycles / rollers/scooters/etc. for transport to the school (including through posters displayed in the school, etc.) /

Organization of events such as "walking bus" (Organization of meeting points, similar to bus stops, to gather students to continue walking to the school) or "day of cycling to school";

Yes/No</td></tr>
<tr><td>Original</td><td>D. SCHOOL FACILITIES</td></tr>
<tr><td>Forward</td><td>D. Facilitățile școlii</td></tr>
<tr><td>Backward</td><td>D. School facilities</td></tr>
<tr><td>Original</td><td>18. Do the majority of students at your school have regular access to any of the following during school hours*?
*During school hours means from the first bell to the last bell, including both instructional and non-instructional time (e.g., lunch).
<table>
<tr><th></th><th>Yes, on grounds only</th><th>Yes, off grounds only</th><th>Yes, both on and off grounds</th><th>No</th><th>Don't know</th></tr>
<tr><td>a. Gymnasium</td><td>☐</td><td>☐</td><td>☐</td><td>☐</td><td>☐</td></tr>
<tr><td>b. Other large room suitable for physical activity (e.g., auditorium, cafeteria, dance studio)</td><td>☐</td><td>☐</td><td>☐</td><td>☐</td><td>☐</td></tr>
<tr><td>c. Fitness room for aerobic and/or strength training</td><td>☐</td><td>☐</td><td>☐</td><td>☐</td><td>☐</td></tr>
<tr><td>d. Running track</td><td>☐</td><td>☐</td><td>☐</td><td>☐</td><td>☐</td></tr>
<tr><td>e. Outdoor sports field (e.g., football or soccer)</td><td>☐</td><td>☐</td><td>☐</td><td>☐</td><td>☐</td></tr>
<tr><td>f. Outdoor paved area (e.g., tennis courts, basketball courts, any paved area that can be used for active games like skipping or hopscotch)</td><td>☐</td><td>☐</td><td>☐</td><td>☐</td><td>☐</td></tr>
<tr><td>g. Skating rink/arena</td><td>☐</td><td>☐</td><td>☐</td><td>☐</td><td>☐</td></tr>
<tr><td>h. Indoor swimming pool</td><td>☐</td><td>☐</td><td>☐</td><td>☐</td><td>☐</td></tr>
<tr><td>i. Secure change room lockers available for use during physical activity</td><td>☐</td><td>☐</td><td>☐</td><td>☐</td><td>☐</td></tr>
<tr><td>j. Change rooms available for use before and after physical activity</td><td>☐</td><td>☐</td><td>☐</td><td>☐</td><td>☐</td></tr>
<tr><td>k. Showers available for use before or after physical activity</td><td>☐</td><td>☐</td><td>☐</td><td>☐</td><td>☐</td></tr>
<tr><td>l. Bicycle racks</td><td>☐</td><td>☐</td><td>☐</td><td>☐</td><td>☐</td></tr>
<tr><td>m. <u>If yes</u>, are the racks in a secure area to avoid theft?</td><td>☐</td><td>☐</td><td>☐</td><td>☐</td><td>☐</td></tr>
<tr><td>n. Grassy playground area</td><td>☐</td><td>☐</td><td>☐</td><td>☐</td><td>☐</td></tr>
<tr><td>o. Playground equipment (e.g., climbing structures, swings)</td><td>☐</td><td>☐</td><td>☐</td><td>☐</td><td>☐</td></tr>
<tr><td>p. Art room</td><td>☐</td><td>☐</td><td>☐</td><td>☐</td><td>☐</td></tr>
<tr><td>q. Music room</td><td>☐</td><td>☐</td><td>☐</td><td>☐</td><td>☐</td></tr>
</table></td></tr>
<tr><td>Forward</td><td>21. La care dintre următoarele facilități au acces elevii școlii dumneavoastră în timpul programului de cursuri (inclusiv în timpul orelor de sport)?

Sală de sport / Alte săli sau spații largi care să permită activitatea fizică (sală de dans, sală de festivități, amfiteatru etc.) / Pistă de alergat / Teren de sport exterior (fotbal, baschet etc.) / Zonă pavată destinată altor activități fizice (șotron, sărit coarda etc.) / Pistă de role / Piscină interioară / Zonă de vestiar securizată utilizabilă pe parcursul activității fizice / Dușuri disponibile înainte și după activitate fizică / Rastel de biciclete într-o zonă care să evite furtul / Zonă acoperită de gazon / Loc de joacă cu echipamente fixe (tobogan, leagăne, masă de ping-pong etc.) / Sală pentru activități artistice (pictură, sculptură etc.) / Sală de muzică;

Da / Nu</td></tr>
<tr><td>Backward</td><td>21. Which of the following facilities do your school's students have access to during school hours (including sports classes)?

Gym / other large halls or spaces that allow physical activity (dance hall, celebration hall, amphitheatre, etc.) / running track / Outdoor Sports Ground (football, basketball, etc.) / paved area for other physical activities (hopscotch, jumping rope, etc.) / roller track / indoor pool /</td></tr>
</table>

<table>
<tr><td></td><td>secure locker area to use before and after physical activity / showers available before and after physical activity / bicycle rack in a secure area / lawn covered area / playground with fixed equipment (slide, swings, ping-pong table, etc.) / room for artistic activities (painting, sculpture, etc.) / music room;

Yes / No</td></tr>
<tr><td>Original</td><td>20. Outside of school hours*, does your school permit regular student access to the following?
*Outside of school hours means before and/or after school, evenings and weekends. Student access may occur via school-led, community-led or informal use.
<table><tr><th></th><th>Yes</th><th>No</th><th>Don't know</th><th>N/A</th></tr><tr><td>a. Gymnasium</td><td>☐</td><td>☐</td><td>☐</td><td>☐</td></tr><tr><td>b. Indoor facilities</td><td>☐</td><td>☐</td><td>☐</td><td>☐</td></tr><tr><td>c. Outdoor facilities (e.g., playing fields, paved activity areas, baseball diamond)</td><td>☐</td><td>☐</td><td>☐</td><td>☐</td></tr><tr><td>d. Equipment (e.g., basketballs)</td><td>☐</td><td>☐</td><td>☐</td><td>☐</td></tr></table></td></tr>
<tr><td>Forward</td><td>22. La care dintre următoarele facilități ale școlii au acces elevii școlii dumneavoastră în afara orelor de curs?

Sală de sport / Sălile aflate în interiorul clădirii / Facilități exterioare (ex. teren de baschet etc.) / Echipamente sportive (ex. mingi de baschet, etc.);

Da/Nu</td></tr>
<tr><td>Backward</td><td>22. Which of the following school facilities do your school's students have access to outside of school hours?

Gym / halls located inside the building / outdoor facilities (ex. basketball court, etc.) / sports equipment (ex. basketballs, etc.);

Yes/No</td></tr>
<tr><td>Original</td><td>21. Outside of school hours*, does your school allow community groups to use the school facilities?
*Outside of school hours means before and/or after school, evenings and weekends.

Yes ☐ No ☐ Don't know ☐</td></tr>
<tr><td>Forward</td><td>23. Permite școala dumneavoastră acces în afara orelor de curs grupurilor organizate pentru utilizarea facilităților școlii destinate activității fizice?

Da / Nu</td></tr>
<tr><td>Backward</td><td>23. Does your school allow before or after-school access to organized groups for the use of physical activity facilities?

Yes / No</td></tr>
<tr><td>Original</td><td>19. Do students have access to the following facilities where they can buy foods or drinks?
<table><tr><th></th><th>Yes</th><th>No</th></tr><tr><td>a. Cafeteria</td><td>☐</td><td>☐</td></tr><tr><td>b. School shop</td><td>☐</td><td>☐</td></tr><tr><td>c. Shops/fast food restaurants close to school</td><td>☐</td><td>☐</td></tr><tr><td>d. Candy and potato chips vending machine</td><td>☐</td><td>☐</td></tr><tr><td>e. Drinks vending machine (e.g., coke, soft drinks, orange juice)</td><td>☐</td><td>☐</td></tr><tr><td>f. Milk vending machine/ milk program (e.g., milk, chocolate milk)</td><td>☐</td><td>☐</td></tr></table></td></tr>
</table>

<table>
<tr><td>Forward</td><td>24. La care dintre următoarele facilități au acces elevii școlii dumneavoastră pentru procurarea de alimente?

Cantină / Magazin în interiorul școlii / Magazine în proximitatea școlii / Restaurante fast-food în proximitatea școlii / Aparate/tonomate cu dulciuri sau chips-uri din cartofi sau alte gustări / Aparate/tonomate cu băuturi / Program de tip „Laptele și cornul” - doar la clasele la care se aplică;

Da/Nu</td></tr>
<tr><td>Backward</td><td>24. Which of the following facilities do your school's students have access to for food procurement?

Cafeteria / shop inside the school / shops near the school / fast food restaurants near the school / vending machines with sweets or potato chips or other snacks / vending machines with drinks / Program such as milk and breadstick” - only for the grades to which it applies;

Yes/No</td></tr>
<tr><td>Original</td><td>E. HEALTHY EATING</td></tr>
<tr><td>Forward</td><td>E. Alimentația sănătoasă</td></tr>
<tr><td>Backward</td><td>E. Healthy Eating</td></tr>
<tr><td>Original</td><td>22. Does your school provide any of the following to promote the sale of healthy food? (Check all that apply)
<table>
<tr><th></th><th>Cafeteria</th><th>Snack bar/ School shop</th><th>Vending machine(s)</th></tr>
<tr><td>a. Healthy food choices at a reasonable/subsidized price</td><td>☐</td><td>☐</td><td>☐</td></tr>
<tr><td>b. Healthy eating promotional materials (e.g., posters)</td><td>☐</td><td>☐</td><td>☐</td></tr>
<tr><td>c. Daily healthy eating specials</td><td>☐</td><td>☐</td><td>☐</td></tr>
<tr><td>d. Healthy eating cafeteria program (e.g., Eat Smart or independent program)</td><td>☐</td><td>☐</td><td>☐</td></tr>
</table></td></tr>
<tr><td>Adapted</td><td>(removed)</td></tr>
<tr><td>Original</td><td>23. Does your school ensure that all students, regardless of ability to pay, have access to fruits and vegetables?
☐ Yes, entire school year
☐ Yes, occasional/short term
☐ No</td></tr>
<tr><td>Adapted</td><td>(removed)</td></tr>
<tr><td>Original</td><td>24. Does your school offer any of the following? (Check all that apply)
☐ Cooking classes
☐ Gardening (e.g., growing produce)
☐ Field trips to farms/farmers’ markets
☐ Media literacy on special topics related to healthy eating (e.g., body image, eating disorders)
☐ Field trips to the local grocery store</td></tr>
<tr><td>Forward</td><td>25. Sunt disponibile vreuna dintre următoarele în școala dumneavoastră? Marcați toate răspunsurile care corespund.

Cursuri de gătit / Activități de cultivare a legumelor și fructelor / Excursii la producători locali de alimente / Informații scrise privind alimentația sănătoasă</td></tr>
<tr><td>Backward</td><td>25. Are any of the following available in your school? Mark all the answers that correspond.

Cooking classes / vegetable and fruit growing / trips to local food producers / written information on healthy eating</td></tr>
</table>

Original	**25. During the past 12 months, did your school initiate/continue any of the following activities/programs at your school?**<br>*Yes* / *No* / *N/A*<br>a. Offered healthy food choices during breakfast program ☐ ☐ ☐<br>b. Offered healthy food choices during lunch program ☐ ☐ ☐<br>c. Offered healthy food choices in the cafeteria(s) ☐ ☐ ☐<br>d. Offered healthy food choices in the snack bar/school shop(s) ☐ ☐ ☐<br>e. Offered healthy food choices in the vending machine(s) ☐ ☐ ☐<br>f. Organized Nutrition Month activities ☐ ☐ ☐<br>g. Stopped the sale of junk food ☐ ☐ ☐<br>h. Held junk food free days ☐ ☐ ☐<br>i. Stopped the sale of sugar-sweetened beverages ☐ ☐ ☐
Forward	26. Pe parcursul **ultimelor 12 luni** a organizat școala dumnevoastră vreuna dintre următoarele activități?<br>*Oferit variante alimentare sănătoase la cantina / Oferit variante alimentare sănătoase la magazinul școlii / Oferit variante alimentare sănătoase la aparatele/tonomatele de alimente sau băuturi / Organizat activități cu scop informativ pentru nutriția sănătoasă / Organizat de activități tip „ziua fără băuturi îndulcite”*<br>*Nu se aplică / Da / Nu*
Backward	26. During the **last 12 months**, has your school organized any of the following activities?<br>*Offered healthy food options at the cafeteria / offered healthy food options at the school store / offered healthy food options at the food or beverage vending machines / organized informative activities for Healthy Nutrition / organized activities such as "day without sweetened drinks"*<br>*Not applicable / Yes / No*
Original	**F. NEIGHBORHOOD/COMMUNITY**
Forward	**F. Împrejurimile școlii**
Backward	**F. School Surroundings**
Original	**26. During the past 12 months, have any of the following items been sold as part of fundraising for any school organization?**<br>*Yes* / *No* / *N/A*<br>a. Chocolate candy ☐ ☐ ☐<br>b. Other candy ☐ ☐ ☐<br>c. Other junk food (e.g., popcorn) ☐ ☐ ☐<br>d. Soda pop or fruit drinks that are not 100% juice ☐ ☐ ☐<br>e. Sports drinks ☐ ☐ ☐<br>f. Cookies, crackers, cakes, pastries, or other baked goods that are not low in fat ☐ ☐ ☐<br>g. Fruits or vegetables ☐ ☐ ☐<br>h. 100% fruit juice or vegetable juice ☐ ☐ ☐<br>i. Low-fat cookies, crackers, cakes, pastries, or other low-fat baked goods ☐ ☐ ☐
Adapted	*(removed)*

<table>
<tr><td>Original</td><td>
27. How much of a problem are the following in the neighborhood where this school is located?
<table>
<tr><th></th><th>Major problem</th><th>Moderate problem</th><th>Minor problem</th><th>Not a problem</th><th>I don't know</th></tr>
<tr><td>a. Tensions based on racial, ethnic, or religious differences</td><td>☐</td><td>☐</td><td>☐</td><td>☐</td><td>☐</td></tr>
<tr><td>b. Garbage, litter, or broken glass in the street or road, on the sidewalks, or in yards</td><td>☐</td><td>☐</td><td>☐</td><td>☐</td><td>☐</td></tr>
<tr><td>c. Selling or using drugs or excessive drinking in public</td><td>☐</td><td>☐</td><td>☐</td><td>☐</td><td>☐</td></tr>
<tr><td>d. Gangs</td><td>☐</td><td>☐</td><td>☐</td><td>☐</td><td>☐</td></tr>
<tr><td>e. Heavy traffic</td><td>☐</td><td>☐</td><td>☐</td><td>☐</td><td>☐</td></tr>
<tr><td>f. Vacant or shabby houses and buildings</td><td>☐</td><td>☐</td><td>☐</td><td>☐</td><td>☐</td></tr>
<tr><td>g. Crime in the neighborhood</td><td>☐</td><td>☐</td><td>☐</td><td>☐</td><td>☐</td></tr>
</table>
</td></tr>
<tr><td>Forward</td><td>27. Cât de mult apreciați a fi o problemă următoarele aspecte legate de zona în care este situată școala?

Tensiuni bazate pe diferențe etnice, religioase sau regionale / Gunoaie aruncate în împrejurimile școlii / Comercializarea de băuturi alcoolice în împrejurimile școlii / Uzul sau tranzacția de droguri în împrejurimile școlii / Bande/găști de cartier / Trafic rutier intens / Clădiri abandonate sau avariate / Rata criminalității din cartier

Problemă majoră / Problemă moderată / Problemă minoră / Nu e o problemă</td></tr>
<tr><td>Backward</td><td>27. How much of a problem are the following issues in the area where the school is located?

Tensions based on ethnic, religious or regional differences / garbage dumped on the school surroundings / sale of alcoholic beverages in the vicinity of the school / use or dealing of drugs in the vicinity of the school / gangs/neighborhood gangs / heavy road traffic / abandoned or damaged buildings / crime rate in the neighborhood

Major problem / moderate problem / minor problem / not a problem</td></tr>
</table>
